# An insight into the role of the N-terminal domain of *Salmonella* CobB in oligomerization and Zn^2+^ mediated inhibition of the deacetylase activity

**DOI:** 10.3389/fmolb.2024.1345158

**Published:** 2024-03-13

**Authors:** Shibangini Beura, Pulak Pritam, Ajit Kumar Dhal, Arindam Jana, Aiswarya Dash, Pritisundar Mohanty, Alok Kumar Panda, Rahul Modak

**Affiliations:** ^1^ Infection and Epigenetics Laboratory, School of Biotechnology, Kalinga Institute of Industrial Technology (KIIT), Bhubaneswar, Odisha, India; ^2^ Environmental Science Laboratory, School of Applied Sciences, Kalinga Institute of Industrial Technology (KIIT), Bhubaneswar, Odisha, India; ^3^ School of Biotechnology, Kalinga Institute of Industrial Technology (KIIT), Bhubaneswar, Odisha, India

**Keywords:** *Salmonella* nicotinamide adenine dinucleotide-dependent deacetylase (CobB), Zn^2+^–CobB interaction, CobB oligomerization, CobB homology modeling, inhibition of CobB deacetylase activity, CobB thermal stability

## Abstract

Prokaryotic deacetylases are classified into nicotinamide adenine dinucleotide (NAD^+^)-dependent sirtuins and Zn^2+^-dependent deacetylases. NAD^+^ is a coenzyme for redox reactions, thus serving as an essential component for energy metabolism. The NAD^+^-dependent deacetylase domain is quite conserved and well characterized across bacterial species like CobB in *Escherichia coli* and *Salmonella*, Rv1151c in *Mycobacterium*, and SirtN in *Bacillus subtilis*. *E. coli* CobB is the only bacterial deacetylase with a known crystal structure (PDB ID: 1S5P), which has 91% sequence similarity with *Salmonella* CobB (SeCobB). *Salmonella* encodes two CobB isoforms, SeCobB_S_ and SeCobB_L_, with a difference of 37 amino acids in its N-terminal domain (NTD). The hydrophobic nature of NTD leads to the stable oligomerization of SeCobB_L_. The homology modeling-based predicted structure of SeCobB showed the presence of a zinc-binding motif of unknown function. Tryptophan fluorescence quenching induced by ZnCl_2_ showed that Zn^2+^ has a weak interaction with SeCobB_S_ but higher binding affinity toward SeCobB_L_, which clearly demonstrated the crucial role of NTD in Zn^2+^ binding. In the presence of Zn^2+^, both isoforms had significantly reduced thermal stability, and a greater effect was observed on SeCobB_L_. Dynamic light scattering (DLS) studies reflected a ninefold increase in the scattering intensity of SeCobB_L_ upon ZnCl_2_ addition in contrast to an ∼onefold change in the case of SeCobB_S_, indicating that the Zn^2+^ interaction leads to the formation of large particles of SeCobB_L_. An *in vitro* lysine deacetylase assay showed that SeCobB deacetylated mammalian histones, which can be inhibited in the presence of 0.25–1.00 mM ZnCl_2_. Taken together, our data conclusively showed that Zn^2+^ strongly binds to SeCobB_L_ through the NTD that drastically alters its stability, oligomeric status, and enzymatic activity *in vitro*.

## Graphical Abstract

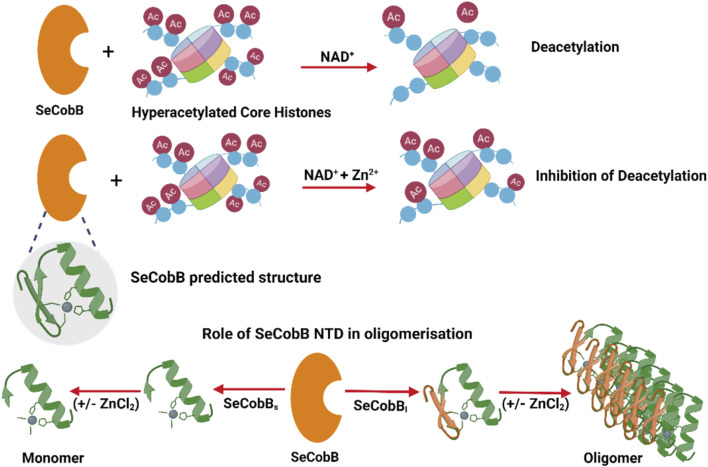

## 1 Introduction

The sirtuin-dependent protein deacylation system in prokaryotes is known to have a significant influence on bacterial physiology by the regulation of gene expression (Lima et al., 2011), maintenance of energy homeostasis (Chan et al., 2011), and modulation of acetate–glucose metabolism by restoring the activity of acetyl-coenzyme A synthetase (Acs) (Castano-Cerezo et al., 2011; Castano-Cerezo et al., 2015). Acetylation can be either enzyme-mediated or non-enzymatic, whereas deacetylation is always an enzyme-mediated process (AbouElfetouh et al., 2015). Prokaryotic deacetylases can be classified into two groups: nicotinamide adenine dinucleotide (NAD^+^)-dependent sirtuins and Zn^2+^-dependent deacetylases (Frye, 2000; Gregoretti et al., 2004). NAD^+^-dependent deacetylases are well studied in both Gram-negative and Gram-positive bacteria like CobB in *E. coli* (de Diego Puente et al., 2015), *Salmonella* (Tucker and Escalante-Semerena, 2010), *Vibrio cholerae* (Liimatta et al., 2018), and *Yersinia pestis* (Liu et al., 2018), Rv1151c in *Mycobacterium* (Liu et al., 2014), SirtA in *Streptomyces* (VanDrisse and Escalante-Semerena, 2018), and SirtN in *B. subtilis* (Gardner and Escalante-Semerena, 2009). Multiple sequence alignments of CobB homologs in selective gastrointestinal bacteria demonstrate 70%–80% sequence homology and a conserved NAD^+^-binding domain [(Mishra et al., 2022), Supplementary Figure S1]. They play diverse roles in various bacterial systems such as regulation of the TacT–TacA toxin–antitoxin system (VanDrisse et al., 2017) and PhoP–PhoQ two-component system in *Salmonella* (Ren et al., 2016), biofilm formation in *Mycobacterium tuberculosis*, resistance to a first-line drug (isoniazid) in *M. smegmatis* (Gu et al., 2015), chemotaxis in *Y. pestis*, and growth homeostasis in *B. subtilis* (Gardner and Escalante-Semerena, 2009). *Salmonella* and other members of the Enterobacteriaceae family encode two CobB isoforms, CobBs (236 aa) and CobB_L_ (273 aa), with a difference of 37 amino acids in its N-terminal. Both the isoforms (SeCobB_S_ and SeCobB_L_) are functional deacetylases, with SeCobB_S_ being enzymatically more active (Tucker and Escalante-Semerena, 2010). *E. coli* CobB (EcCobB, PDB ID: 1S5P) is the only bacterial deacetylase whose crystal structure is solved so far, which displays 91% sequence similarity with *Salmonella* CobB (SeCobB). Our homology modeling and the AlphaFold structure database showed that like EcCobB, *Salmonella* CobB also contains a zinc-binding motif with unknown function.

Zinc acquisition and homeostasis contribute significantly toward bacterial physiology and pathogenesis (Nairz et al., 2010; Ammendola et al., 2016). This homeostasis is crucial not only for the expression of metallozymes (Hood and Skaar, 2012) and other proteins related to bacterial metabolism but also for the adequate expression of virulent factors to cause infection (Waldron and Robinson, 2009; Vickers, 2017). Among micronutrients like copper, zinc, manganese, and iron, maintaining the intracellular and extracellular levels of zinc is of utmost importance for the structural and catalytic regulation of various bacterial proteins involved in processes like DNA replication and oxidative stress response (Cerasi et al., 2013). This regulation is achieved by zinc efflux and influx transporters like P1B-type ATPase ZntA and ZnuABC in *E. coli*, respectively (Hazan et al., 2001; Wei and Fu, 2006; Romiguier and Roux, 2017). Zinc serves as a crucial bridge between bacterial metabolism and defense mechanisms against the host in *Salmonella* by ZnuABC, a zinc uptake transporter. Zn metal has a provident impact on the regulation of the protein structure and function due to its strong affinity toward amino acid residues, especially cysteine (Tainer et al., 1991; Giles et al., 2003). Zn metal–cysteine complexes have multifaceted functions like the inhibition of enzymatic activity in dimethylarginine dimethylaminohydrolase (DDAH-1) (Maret, 2013a), function as a redox switch in betaine–homocysteine methyltransferase (BHMT) (Maret, 2005), and act as a stabilizing bridge between protein complexes like in endothelial NOS isoform (NOS3) (Fischmann et al., 1999).

Here, we report that SeCobB_L_ is an oligomeric protein, whereas SeCobB_S_ is a monomer in solution, which clearly indicates that oligomerization is mediated through a 37-amino acid N-terminal domain (NTD). Zn^2+^ binds to both isoforms, SeCobB_L_ and SeCobB_S_, albeit with different affinities. SeCobB_S_ has weak binding affinity for Zn^2+^, which indicates a very weak interaction with the predicted Zn-binding motif. SeCobB_L_ strongly binds to Zn^2+^, which is presumably mediated through the NTD. The SeCobB–Zn^2+^ interaction greatly enhances the kinetic and thermal stability of both the proteins in the solution. Dynamic light scattering (DLS) showed that ZnCl_2_ induces a ninefold increase in the SeCobB_L_ scattering intensity in the solution compared to the ∼ onefold change in the case of SeCobB_S_, which indicated that Zn^2+^ induced the formation of larger particles of SeCobB_L_. Both SeCobB_S_ and SeCobB_L_ deacetylase activities are inhibited at a higher concentration of Zn^2+^. Taken together, our study is the first report to demonstrate the function of NTD of SeCobB_L_ and effect of Zn^2+^ on the stability and activity of SeCobB. We also show that the predicted Zn-binding domain plays a limited role in Zn^2+^ binding *in vitro*, and we predict that similar effects will be observed in SeCobB homologs.

## 2 Materials and methods

### 2.1 Bacterial strains, plasmids, and culture conditions

The bacterial strains used in this study are *E. coli* DH5α and *E. coli* BL21 codon plus (DE3). These strains were grown in LB broth at 37°C, 150 rpm. Antibiotics were added as required for the culture at the indicated concentration—chloramphenicol (20 μg ml^−1^), tetracycline (20 μg ml^−1^), and kanamycin (50 μg ml^−1^). Both the larger and shorter isoforms of the SeCobB gene were cloned using genomic DNA of *Salmonella enterica* subspecies I serovar Enteritidis str. P125109. The pET 28a (+) plasmid was used for both cloning and recombinant protein expression. The detailed protocol for cloning is given in Supplementary Material.

### 2.2 Purification and characterization of SeCobB

Both shorter and full-length isoforms of the SeCobB protein (SeCobB_S_ and SeCobB_L_, respectively) were purified using Ni^2+^–NTA affinity chromatography (Supplementary Figure S2). The oligomeric status of SeCobB isoforms was determined by size exclusion chromatography (SEC) using a Sephacryl S-200 16/60 GPC column attached to AKTA Pure (GE Healthcare). The column was pre-equilibrated with a protein elution buffer (25 mM Tris pH 8, 200 mM NaCl, 2 mM β-mercaptoethanol, and 5% glycerol) at a flow rate of 0.5 ml/min. It was calibrated using SEC standard protein markers (Gel Filtration Markers Kit, Sigma-Aldrich: MWGF200), and the void volume (Vo) was determined by passing blue dextran under the same conditions. Each of the eluted peaks were analyzed by SDS-PAGE to identify the presence of SeCobB_S_ and SeCobB_L_. The apparent molecular weight of SeCobB_S_ and SeCobB_L_ was determined by interpolating the peak elution volume (Gupta et al., 2000) on the SEC standard plot. The SEC standard plot is a linear calibration curve produced by plotting the logarithms of the known molecular masses (log MW) of protein standards *versus* their respective Ve/Vo values, where Ve is the elution volume and Vo is the void volume.

The secondary structure of recombinant SeCobB_S_ and SeCobB_L_ was determined using circular dichroism (McDevitt et al., 2011) spectra using a Chirascan CD spectrometer (Applied Photophysics) at the Central Research Facility of Institute of Life Sciences, Bhubaneswar, India. A graph was plotted between wavelength (λ) and molar ellipticity (ϴ) after baseline correction. All the data are represented as an average of three scans, with the protein purified in three independent batches.

### 2.3 Solution-state structures of SeCobB_S_ and SeCobB_L_ obtained using DLS

To gain insights into how the solution-state structure of the protein changes in the presence of a divalent metal, DLS experiments were conducted using a multi-angle particle size analyzer from Photocore Ltd. (Russia). DLS was utilized to determine the size distribution of the protein in both the absence and presence of ZnCl_2_.

Ni–NTA-purified recombinant SeCobB in the protein elution buffer (25 mM Tris, pH 8, 200 mM NaCl, 2 mM β-mercaptoethanol, and 5% glycerol) was subjected to high-speed centrifugation at 12,000 rpm, 10 min at 4°C to avoid any possible air bubbles. The supernatant was used to prepare suitable dilutions using the protein elution buffer as the solvent. They were transferred to a clean and dry cylindrical glass vial of 10 mm diameter for carrying out DLS. The outer surface of the cuvette was gently wiped with lint-free tissue before placing into the instrument to remove dust or dirt to avoid unnecessary scattering from the glass wall.

In the DLS study, the protein suspension at a very dilute concentration is illuminated with a laser light of wavelength (λ) 654 nm, and the scattering intensity is collected at a scattering angle θ (=90^o^) using a photon detector. The intensity auto-correlation is calculated as (Lima et al., 2011)

$$g^{1}\left(\tau \right)=\sqrt{\frac{\left.{\left(g\right.}^{2}-1\right)}{\beta }}.$$ (1)

In Eq. 2, *I(t)* is the correlation function at time *t* and $I\left(t+\tau \right)$ is the correlation of the same signal with a delay time. The angular brackets denote the average over time *t*. The intensity auto-correlation is related to the field correlation as (Lima et al., 2011)

$$g^{2}\left(t\right)=\frac{<I\left(t\right)I\left(t+\tau \right)>}{<I\left(t\right){>}^{2}}.$$ (2)

**FIGURE 1:**
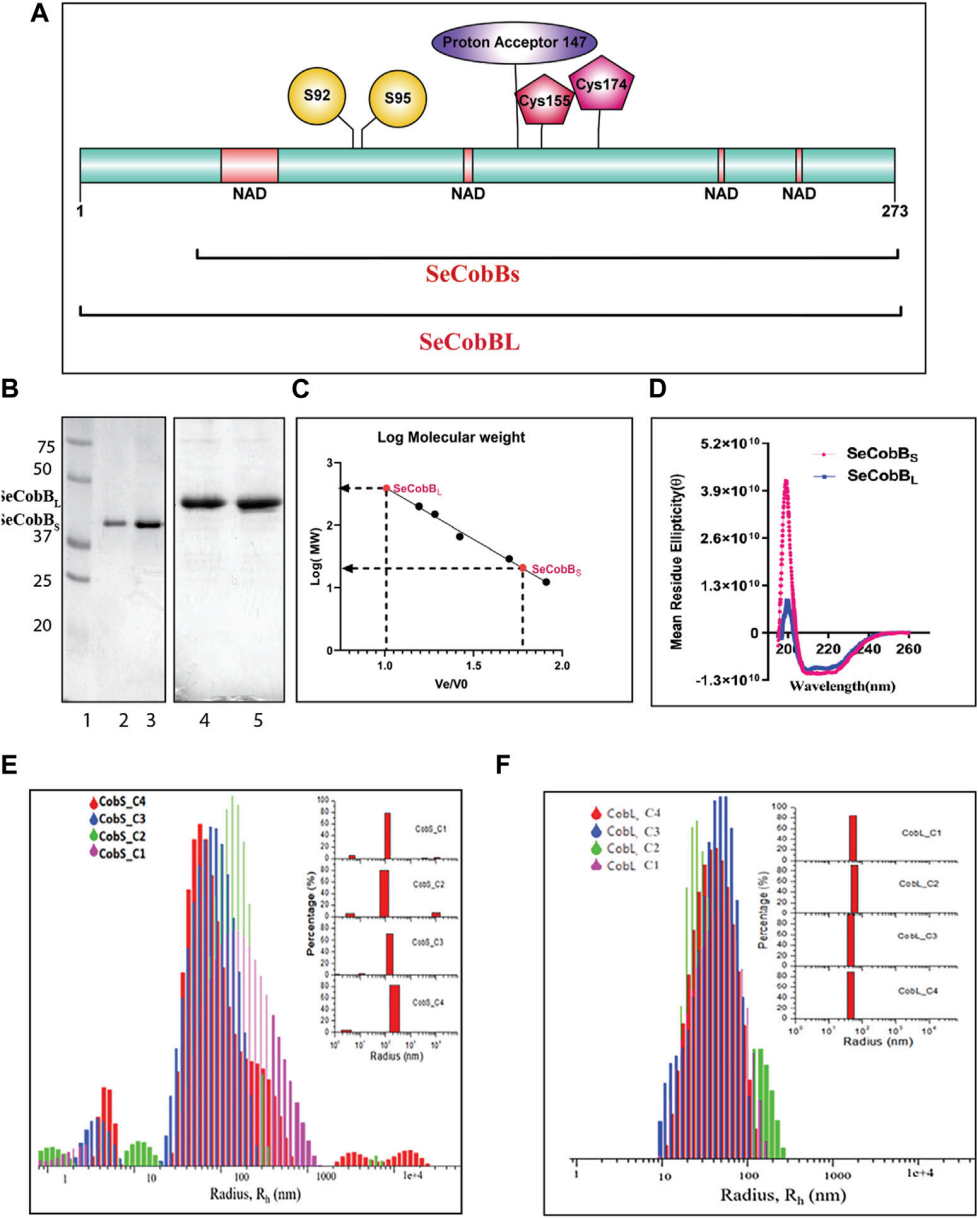
Characterization of the *S. enterica* deacetylase protein isoforms, SeCobB_S_ and SeCobB_L_. **(A)** Domain organization of the *S. enterica* CobB protein. SeCobB_S_ lacks the initial 37 amino acid residues present in SeCobB_L_. The predicted domains are represented in different colors: NAD^+^-binding domain in peach, Zn^2+^-binding sites in maroon (Cys 155 and Cys 174), substrate-binding site in yellow (S92 and S95), and active site in purple (147). The full-length protein is represented in cyan. (1–273). **(B)** SeCobB protein purification profile. Lane 1—Bio-Rad protein ladder; lanes 2 and 3—purified SeCobB_S_ at 30 kDa; lanes 4 and 5—purified SeCobB_L_ at 34 KDa. **(C)** Size exclusion chromatography profile of SeCobB_S_ and SeCobB_L_ using a HiPrep 16/60 Sephacryl S-200 column. The standard curve was generated by plotting Ve/Vo vs. log of molecular weight of the SEC standards (Ve—elution volume of each protein; Vo—void volume). The solution molecular weight of SeCobB_S_ and SeCobB_L_ was determined by interpolating the Ve/Vo value on the standard curve (dotted line). **(D)** Determination of secondary structures of SeCobB_S_ and SeCobB_L_ using circular dichroism spectroscopy. Molar residue ellipticity (θ) values plotted against the wavelength (λ) showed two peaks at 208 and 222 nm. Size distribution plots of hydrodynamic radius R_h_ (in nm) of recombinant SeCobB_S_ **(E)** and SeCobB_L_ **(F)** at different concentrations. The concentrations CobS_C1–CobS_C4 correspond to 93 μg/ml, 187 μg/ml, 375 μg/ml, and 750 μg/ml of the SeCobB_S_ protein, respectively. The concentrations CobL_C1–CobL_C4 correspond to 100 μg/ml, 200 μg/ml, 500 μg/ml, and 800 μg/ml, respectively. The inset bar diagram represents the % size distribution for SeCobB_S_ **(E)** and SeCobB_L_ **(F)** against the radius (nm) of different particle sizes.

The correlation functions are analyzed using a CONTIN-based method, and the corresponding size distributions are obtained by fitting the correlation function with Eq. 2 (see Figure 1).

$$g^{1}\left(\tau \right)=\int_{0}^{\infty }G\left(\Gamma \right)e^{-\Gamma \tau }d\Gamma ,$$ (3)

where Γ is the characteristic decay rate, which relates the translational free diffusion coefficient *D*_o_ as

$$\Gamma =D_{o}Q^{2}.$$ (4)

Here, *Q* is the scattering vector and is related to the scattering angle θ by $Q=\frac{4\pi \mu \;\sin\left(\frac{\theta }{2}\right)}{\lambda }$ , and *D*_o_ is given by the Stokes–Einstein equation:

$$D_{o}=\frac{k_{B}T}{6\pi \eta R_{h}}.$$ (5)

Here, k_B_ is the Boltzmann constant, *T* is the absolute temperature, *η* is the viscosity of the solvent, and *R*_*h*_ is the hydrodynamic radius.

### 2.4 Prediction and optimization of the predicted 3D-modeled structure

The primary sequence of the NAD^+^-dependent deacylase protein of *Salmonella* Enteritidis PT4 (strain P125109) was retrieved from the UniProtKB database (accession ID: A0A6C7HR52); the protein had a length of 273 aa (SeCobB_L_) (Consortium, 2015). However, we deleted the first 37-amino acid (aa) sequence to check their binding affinities against Zn^2+^ in a comparative way, where the 236-aa sequence was referred to as the shorter isoform (SeCobB_S_) and the 273-aa sequence was termed the longer isoform (SeCobB_L_). The three-dimensional (3D) structure of both SeCobB_L_ and SeCobB_S_ was determined using online servers like I-TASSER (Roy et al., 2010). The best I-TASSER-generated structure was selected for refinement based on its C-score value. Qualitative analysis of the best modeled structure of both protein isoforms was done through different online servers.

Servers like PROCHECK (Roman et al., 1993), Verify3D (Lüthy et al., 1992), and ERRAT (Colovos and Yeates, 1993) were used for the quality factor analysis of the model protein from the SAVES meta-server. The ProSA webserver (Wiederstein and Sippl, 2007) was used for the analysis of the Z-score of the target protein. However, the best modeled structure was obtained from the I-TASSER server, with 2.1% residues for SeCobB_L_ and 1.5% residues for SeCobB_S_ in the outliers and 72% in the case of SeCobB_L_ and 74% in the case of SeCobB_S_ in the favorable region. These structures were then selected for refinement based on their respective C-score values in the GalaxyRefine webserver (Ko et al., 2012). This course of action was repeated until the quality of the structural conformation failed to increase any further.

### 2.5 *In vitro* histone deacetylase assay

The *in vitro* histone deacetylase assay was standardized in the laboratory (Mishra et al., 2022). In brief, HCT-116 cells were treated with sodium butyrate (NaBU) for 24 h to hyperacetylate all the proteins including histones in the cells. Mammalian hyperacetylated histones were enriched by TCA precipitation and used as a substrate for HDAC assays. Then, 50–200 ng of recombinant SeCobB was incubated with 10 μg hyperacetylated, acid-extracted core histones in the presence of NAD^+^ (5 mM) as a cofactor in the HDAC assay buffer (50 mM Tris-Cl, 137 mM NaCl, 2.7 mM KCl, 1 mM MgCl_2_, 1 mM DTT, 5% glycerol, and 0.2 mM PMSF) for 60 min at 37°C.

We also performed an *in vitro* histone deacetylase assay in the presence of zinc (ZnCl_2_). A measure of 0.5 M ZnCl_2_ stock was prepared by dissolving 681.45 mg ZnCl_2_ (SRL-87288) in a minimum of 2 N HCl and then increasing the volume up to 10 ml with distilled water. A measure of 100 ng recombinant SeCobB was incubated with 10 μg hyperacetylated, acid-extracted core histones in the presence of NAD^+^ (5 mM), along with varying concentrations of ZnCl_2_ (0.25–2.5 mM) under the same conditions as mentioned above. The immunoblots were probed with anti-acetyl lysine and anti-H3 antibodies. The *in vitro* deacetylase assay for both SeCobB_S_ and SeCobB_L_ was performed separately.

### 2.6 Molecular docking studies

To check the binding mode interaction between Zn^2+^ and SeCobB_S_/SeCobB_L_, docking studies were performed using AutoDock v4.2.6 software (Olson and Olson, 2010). It was initialized with protein and ion preparations. The grid scale was positioned at 40 × 40 × 40 *xyz* points with a grid spacing of 0.375 Å, and the grid core was chosen at dimensions (*x*, *y*, and *z*) 54.535, 58.898, and 50.521, respectively. The docking analysis was carried out using a rigid protein and genetic algorithm with the following default parameters: the maximum number of generations = 2700, maximum number of seeds = 2,500,000 runs, population size of 150, and 100 GA runs. Following that, docking was done by setting the parameters to default values, followed by using the command-line interface for autogrid and autodock applications.

We performed a specific docking approach on both sites and a blind docking approach to check the binding affinity of Zn^2+^ toward SeCobB_S_ using AutoDock Vina software and the online server Metal Ion-Binding site prediction and modeling server (MIB2), respectively. A webserver that was used to create the expected metal ion-bound 3D structure and prediction of metal ion-binding residues was defined. To create a binding template, areas that bind 12 different types of metal ion-binding residues were taken into consideration. The query protein and the template were compared structurally using the fragment transformation approach without any data training. The template contained residues that are within 3.5 Å of the metal ions by modifying the scoring algorithms based on structural and binding residue similarity. The prediction of binding residues for 18 different types of metal ions, namely, Ca^2+^, Cu^2+^, Fe^3+^, Mg^2+^, Mn^2+^, Zn^2+^, Cd^2+^, Fe^2+^, Ni^2+^, Hg^2+^, Co^2+^, Cu^+^, Au^+^, Ba^2+^, Pb^2+^, Pt^2+^, Sm^3+^, and Sr^2+^, is supported. The metal ion docking after prediction is also provided using MIB (Lu et al., 2012; Lin et al., 2016).

### 2.7 Fluorescence quenching studies to determine the SeCobB–Zn^2+^ interaction

SeCobB_S_ and SeCobB_L_ (5 µM in the protein elution buffer, pH 8.0) were titrated with ZnCl_2_ (0–100 μM, at a rate of 5 µM per addition). Tryptophan fluorescence intensity of this bivalent metal ion-bound SeCobB_S_ and SeCobB_L_ was recorded with an excitation wavelength of 295 nm using a spectrofluorometer (FLS1000, Edinburgh Instruments, United Kingdom). The temperature of the samples was maintained at 25°C by using Peltier attached to the spectrofluorometer. Stern–Volmer plots and Scatchard analysis were done using corrected fluorescence data considering the effect of dilution. The linear fit of the data was obtained using the Stern–Volmer equation,

$$\frac{F_{0}}{F}=1+K_{SV}\left[Q\right],$$ (6)

and the Scatchard equation,

$$\log\left[\frac{F_{0}-F}{F}\right]=logK+nlog\left[Q\right],$$ (7)

where *F*_*0*_ and *F* are the emission intensities of SeCobB_S_ and SeCobB_L_ in the absence and presence of the zinc ions, respectively, provided the Stern–Volmer quenching constant (K_SV_), the binding constant (K_b_), and the number of binding sites (n). Here, [Q] stands for [zinc ions].

### 2.8 Effect of Zn^2+^ on temperature-induced unfolding of SeCobB

The thermal stability of SeCobB_S_ and SeCobB_L_ was determined using a thermal-induced denaturation experiment. In brief, both protein isoforms (5 µM in the protein elution buffer, pH 8.0) were incubated in the absence and presence of ZnCl_2_ (0–100 µM). Intrinsic tryptophan fluorescence spectra of all the samples were recorded in the 310–400-nm region using an excitation wavelength of 295 nm. The change in tryptophan fluorescence at 334 nm was recorded stepwise between 25°C and 90°C. As mentioned previously (Nandi et al., 2013), Vant Hoff enthalpy (ΔHVH) and entropy (ΔS) were calculated from the thermal melting data.

## 3 Results

### 3.1 Characterization of the *S. enterica* deacetylase protein SeCobB

The schematic domain organization of SeCobB, illustrating both shorter and larger isoforms, the predicted NAD^+^-binding domain, Zn^2+^-binding site, substrate-binding site, and active site, is shown in Figure 1A. To further characterize *Salmonella* CobB (SeCobB), we cloned it in *E. coli* using a pET-28a (+) vector, and the N-terminal 6×His-tagged protein was extracted and purified by Ni–NTA agarose metal affinity chromatography (Figure 1B, lanes 2–3 and 4–5). Size exclusion chromatography showed that SeCobB_S_ exists as a monomer, whereas SeCobB_L_ exists as an oligomer in solution (Figure 1C). Circular dichroism spectroscopy revealed that both isoforms have a similar secondary structure that predominantly harbors alpha helices, illustrated by peaks at 208 and 222 nm, respectively (Figure 1D).

We determined the oligomeric status of SeCobB isoforms by multi-angle dynamic light scattering. The concentration-dependent correlation function for SeCobB_S_ (denoted as CobS_C1-CobS_C4) and SeCobB_L_ (denoted as CobL_C1-CobL_C4) is shown in Supplementary Figure S3. The data are fitted with the distribution of decay rates G (Γ) using Eq. 2. The experimental function (Eq. 1) agreed well with the theoritical fit function, and the obtained distribution of the hydrodynamic radius (Eq. 5) was plotted as shown in Figures 1E, F for SeCobB_S_ and SeCobB_L_, respectively. It is worth noting that for all concentrations ranging from 93 to 750 μg/ml of SeCobB_S_, the size distribution curve has multiple peaks that vary from 5 nm to several microns, which clearly demonstrates the heterogeneous nature of protein particles with a large variation in their sizes coexisting in the solution state (Figure 1E). SeCobB_L_ gives a single distribution peak that overlaps with each other for the entire range of concentrations (Figure 1F), demonstrating a homogeneous particle size distribution within the protein solution.

The bar diagram representation corresponding to the hydrodynamic radius plot is demonstrated as a function of the percentage (%) of size distribution for SeCobB_S_ and SeCobB_L_ in the inset of Figures 1E, F, respectively. The major contribution (∼75%) in CobS_C1 to CobS_C4 comes from the main peak that corresponds to a mean radius of 147 nm ± 60 nm, whereas the radius of the small peak varies from 2 to 11 nm with a population of ˂ 5% of total proteins in the solution coexisting with a small % of micrometer-sized aggregates. These proteins, which are monomers with a radius range of 2–11 nm, can be aligned to their molecular weight estimated in GPC. In addition, we also observe large aggregates of the protein at a high concentration (CobS_C3 and CoBS_C4) amounting to a small population (<5%). SeCobB_L_, which is an oligomer in solution, displays a single peak corresponding to a mean radius of 49.99 nm ± 15 nm that coincides with the molecular weight estimated in GPC.

### 3.2 *Salmonella* CobB is a NAD^+^-dependent functional protein lysine deacetylase

**FIGURE 2:**
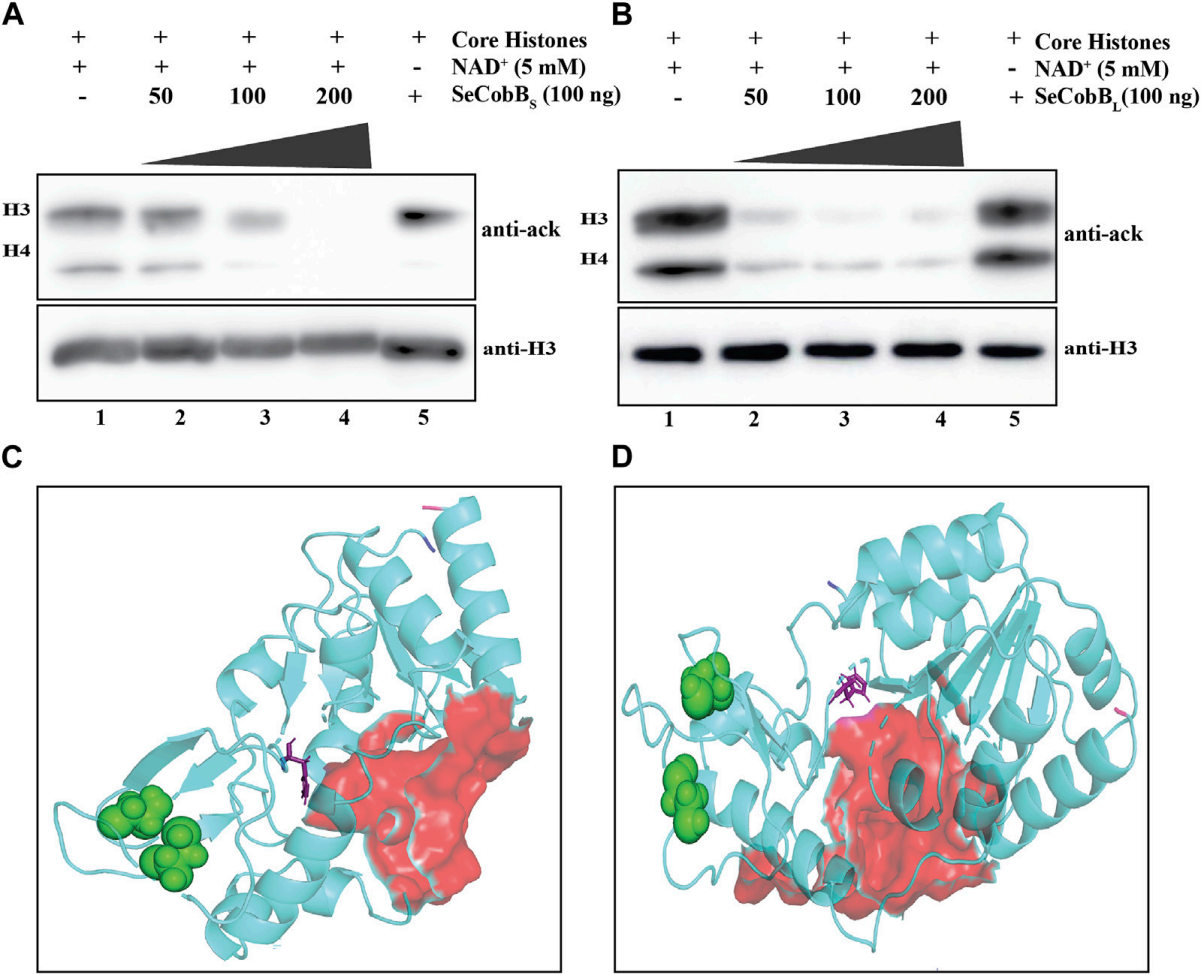
**(A, B)** *In vitro* deacetylase assay to show that SeCobB is a NAD^+^-dependent deacetylase. *In vitro* histone lysine deacetylase assay with SeCobB_S_ **(A)** and SeCobB_L_ **(B)** using hyperacetylated mammalian core histones as the substrate. The upper panel shows Western blotting with the anti-acetyl lysine antibody. Lane 1—acid-extracted core histones (substrate); lanes 2–4—core histones incubated with purified SeCobB_S_ **(A)** and SeCobB_L_ **(B)** at different concentrations (50, 100, and 200 ng), along with NAD^+^ as the cofactor; lane 5—core histones incubated with 200 ng purified enzyme without NAD^+^. Immunoblots were re-probed with the anti-H3 antibody to confirm equal sample loading (bottom panel). The assay was performed in three biological replicates. **(C, D)** Homology modeling to illustrate the predicted structure of SeCobB. Cartoon representation of the modeled structure of SeCobB_S_ **(C)** and SeCobB_L_ **(D)** using the I-TASSER server. The N-terminal and C-terminal ends are shown in blue and hot pink, respectively. The NAD^+^-binding sites are shown as surface-shaped (red), zinc-binding sites (sphere-shaped in green), and the active sites are shown as stick-shaped in purple.

A deacetylase protein of *Archaeoglobus fulgidus*, Sir2-Af2 deacetylating C-terminal of p53 peptide, and yeast Hst2 deacetylating acetylated histone H4 peptide have been reported (Tanny et al., 2004). Thus, we investigated whether SeCobB can deacetylate core histones. To establish the same, we performed *in vitro* histone deacetylase assay on acid-extracted, hyperacetylated core histones with SeCobBs and SeCobB_L_ separately in the presence and absence of NAD^+^, followed by Western blotting with anti-acetyl lysine and anti-H3 antibodies. The acetyl lysine antibody detected the concentration-dependent deacetylation of core histones by both isoforms in the presence of NAD^+^ (Figures 2A, B, lanes 2–4), whereas no deacetylation occurred in the absence of NAD^+^ (lane 5, top panel, Figures 2A, B). A gradual decrease in the band intensity was representative of concentration-dependent deacetylation. An equal amount of substrate was used in each reaction, as indicated by the uniform band intensity of H3 using the anti-H3 antibody (Figures 2A, B, bottom panel).

### 3.3 Three-dimensional structure prediction of SeCobB

No structural information is available for NAD^+^-dependent protein deacylase (CobB) of *Salmonella* Enteritidis PT4 (strain P125109). Due to a lack of validated structural information about the target protein, Zn^2+^-binding site and NAD^+^-binding site residues were predicted using UniProt (Figure 1A) and I-TASSER servers. The I-TASSER-modeled structure of SeCobB_L_ was compared with the AlphaFold database-predicted structure to check their structural similarity. The structural alignment showed that the RMSD between these two structures was 1.549 Å (Supplementary Figure S5), which is less than the default cut-off of RMSD of 2 Å, and that the extent of dissimilarity between two structures were not greater, as per their structural comparison. Likewise, the predicted NAD^+^-binding sites and zinc ion-binding sites are shown in Figure 1. The protein 3D structure generated through the I-TASSER server represents the good quality after validation through Ramachandran plot analysis, Verify3D, ERRAT, and the ProSA server (Tables 3, 4). The Ramachandran plot analysis of the SeCobB_S_ model structure before refinement revealed that 74.0% of residues were in the allowed region and 1.5% in the disallowed region. However, after protein structure refinement using the GalaxyRefine webserver, the number of amino acid residues in the allowed region increased to 92%, and only 1% of residues were in the disallowed area. Similarly, the number of residues in the SeCobB_L_ modeled structure was shifted from 72.0% to 89.8% in the allowed region, whereas it was shifted from 2.1% to 1.7% in the disallowed region. The validation of our selected model through the Verify3D program (checks for the compatibility of a three-dimensional atomic model with its own one-dimensional amino acid sequence) revealed that there were 74.15 residues for SeCobB_S_ and 72.53 residues for SeCobB_L_ with an average 3D–1D score >0.2 (Supplementary Figures S4E, F). The server “ERRAT” provided the overall quality factor (expressed as the percentage of the protein for which the calculated error values fall within the 95% rejection limit) of the model as 93.1818 for SeCobB_S_ and 94.024 for SeCobB_L_ (Supplementary Figures S4C, D). We studied the quality of the target protein using the ProSA webserver, and it reported a Z-score value of −7.05 (SeCobB_S_) and −7.92 (SeCobB_L_), which is well within the range of the native conformation of the crystal structure. The ProSA analysis of the model structure revealed an improvement in the Z-score after structural refinement.

### 3.4 Zn^2+^ has an inhibitory effect on the NAD^+^-dependent *in vitro* deacetylase activity of SeCobB

**FIGURE 3:**
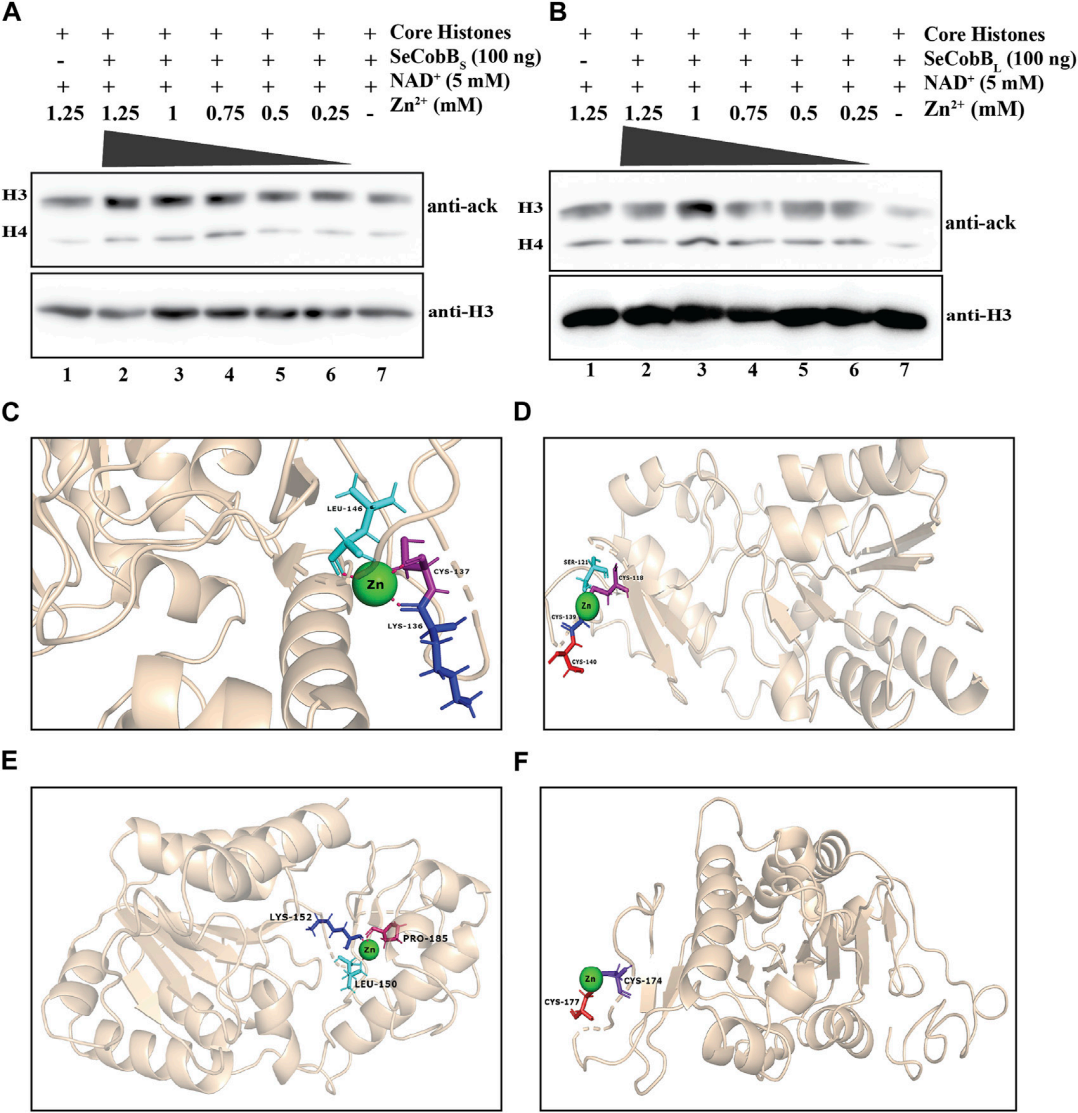
**(A, B)** Effect of Zn^2+^ on SeCobB activity. *In vitro* histone lysine deacetylase assay with 100 ng of purified SeCobB_S_ **(A)**, SeCobB_L_ **(B)**, and ZnCl_2_ using hyperacetylated HCT-116 core histones as the substrate. Immunoblots were probed with pan-acetyl lysine (upper panel) and anti-H3 (bottom panel) antibodies. Lane 1 **(A, B)**—acid-extracted core histones (substrate); lanes 2–7 **(A, B)**—core histones incubated with ZnCl_2_ at different concentrations (1.25, 1, 0.75, 0.5, and 0.25 mM), along with NAD^+^(5 mM) as the cofactor. Immunoblots were re-probed with the anti-H3 antibody to confirm equal loading of the sample. The assay was performed with three independent batches of purified protein. **(C, D)** Molecular docking study to predict the amino acid residues involved in the Zn^2+^–SeCobB_S_ interaction. Cartoon representation of the docked complex of the SeCobB_S_ modeled structure against Zn^2+^ through a site-specific approach using AutoDock Vina **(C)** and **(D)** blind docking approach using the MIB2 webserver. **(E, F)** Molecular docking study to predict the amino acid residues involved in the Zn^2+^–SeCobB_L_ interaction. Cartoon representation of the docked complex of the SeCobB_L_ modeled structure against Zn^2+^ through a site-specific approach using AutoDock Vina **(E)** and **(F)** blind docking approach using the MIB2 webserver. The target protein is shown in wheat, whereas Zn^2+^ (sphere-shaped) (green) and the H-bond residues are stick-shaped.

The predicted 3D model structure of *Salmonella* CobB (Figure 3C) shows that it contains a zinc-binding motif. Most of the Sirt2 family proteins contain a Cys-X-X-Cys-(X)15–20-Cys-X-X-Cys sequence as a characteristic feature of the Zn^2+^-binding motif within their conserved domain. The predicted zinc-binding sites in SeCobB according to “UniProt” software are cysteine at positions 155 and 174 (Figure 1A). To explore the effect of the Zn^2+^–cysteine interaction on the activity of *Salmonella* CobB, we performed an *in vitro* deacetylase assay at different concentrations of ZnCl_2_ with SeCobB_S_ and SeCobB_L_ separately (Figures 3A, B). The anti-acetyl lysine antibody detected a gradual decrease in the band intensity with decreasing ZnCl_2_ concentration, representative of the increase in the deacetylase activity (Figures 3A, B, lanes 2–6). The band intensity was the lowest in the absence of ZnCl_2_, indicative of maximum deacetylation by restoring the deacetylase activity (Figures 3A, B, lane 7). An equal loading of the substrate was confirmed by re-probing the blots with the anti-H3 antibody (Figures 3A, B, bottom panel).

Since a predicted zinc-binding motif exists within SeCobB, whose function is unknown, and an *in vitro* HDAC assay illustrated that zinc mediated inhibition in the deacetylase activity, we performed a docking analysis of Zn^2+^ ions with the predicted 3D model structure of SeCobB_S_ (Figures 3C, D). The predicted Zn^2+^-binding sites in the case of SeCobB_L_ are Cys-155 and Cys-174, while it is Cys-118 and Cys-137 in the case of SeCobB_S_. The site-specific docking study of SeCobB_L_–Zn^2+^ revealed that Zn^2+^ showed three H-bond interactions (Leu-150, Lys-152, and Pro-185) (Figure 3E), none of which is present within the predicted Zn^2+^-binding site. In the case of SeCobB_S_, the H-bond interactions are Lys-136, Cys-137, and Leu-146 (Figure 3C), among which only CYS-137 is present within the predicted Zn^2+^-binding sites. These H-bond interactions showed a binding affinity of −1.2 kcal/mol toward Zn^2+^ for both isoforms. The blind docking study revealed that Zn^2+^ showed two H-bond interactions (Cys-174 and Cys-177) for SeCobB_L_ and four H-bond interactions (Cys-118, Ser-121, Cys-139, and Cys-140) for SeCobB_S_ (Figures 3D, F), of which Cys-174 for SeCobB_L_ and Cys-118 in the case of SeCobB_S_ are the only residues present within the predicted Zn^2+^-binding sites. The hydrogen bond interactions showed a binding score of 3.222 for the longer isoform and 3.272 for the shorter isoform with zinc-binding sites.

### 3.5 Solution structure of SeCobB in the presence of ZnCl_2_

**FIGURE 4:**
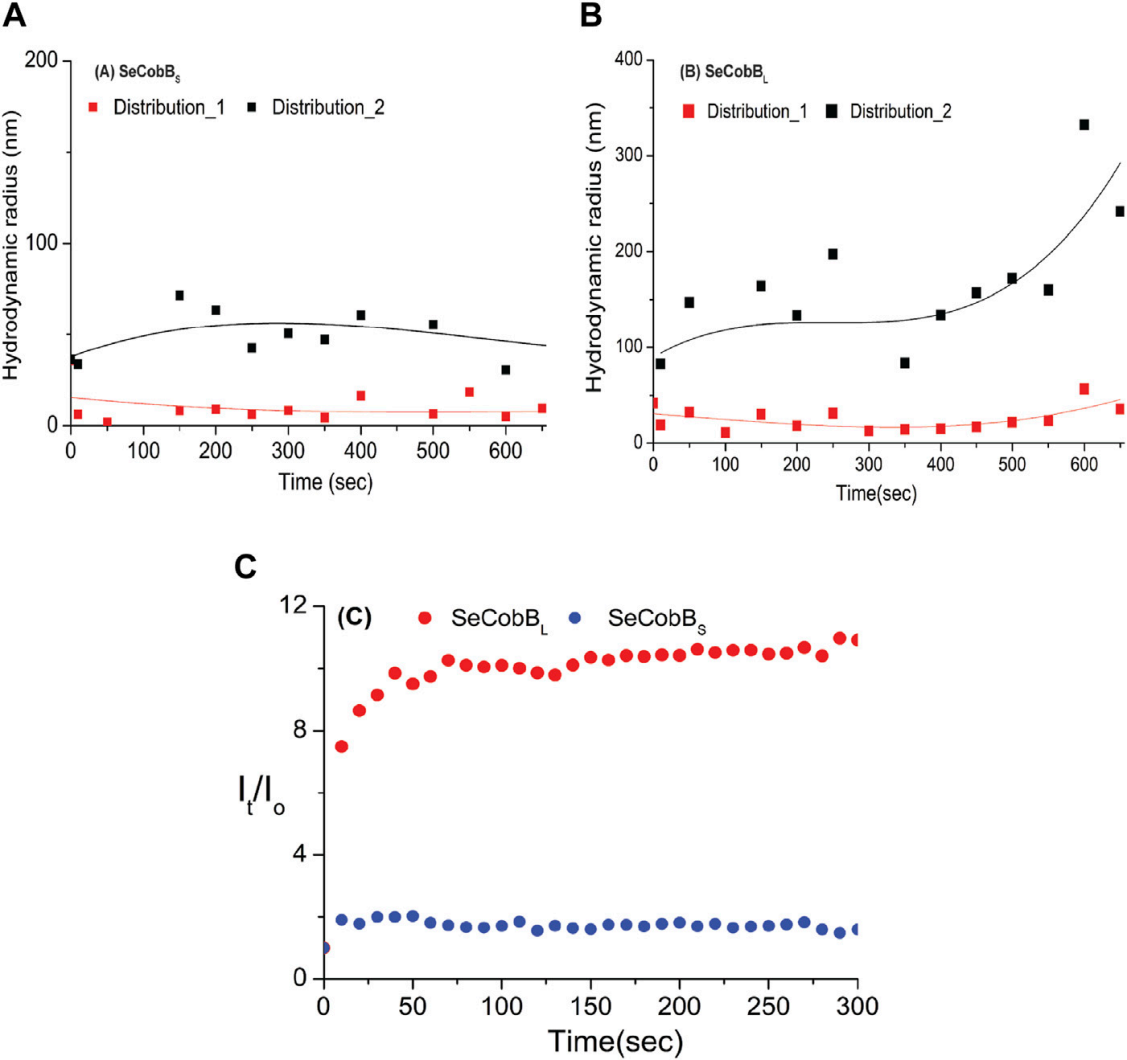
DLS studies (at T = 20°C) were conducted to investigate the kinetics of the solution-state structure of SeCobB_S_ compared to SeCobB_L_ after induction with 250 μM ZnCl_2_ solution. Two distinct types of size distributions, based on hydrodynamic radius, were observed for SeCobB_S_ **(A)** and SeCobB_L_ **(B)**. The time-dependent evolution of the hydrodynamic radius is plotted AutoDock Vina **(A,B)**. The line drawn on the data points in **(A)** and **(B)** is guided to the eye. Additionally, the corresponding average intensity, denoted as I_t_ and normalized to the intensity at t = 0, is plotted as a function of time **(C)**. The experiment was performed in three individual batches.

Divalent cations are known to induce protein aggregation in solution, leading to a change in particle size, which can be studied by DLS and other techniques. This, coupled with fluorescence spectroscopy and other techniques, helps further elucidate the complex interplay of factors that influence a protein’s size and aggregation behavior in the presence of metal ions and salt. In the DLS experiment, the protein solution was treated with 250 μM ZnCl_2_, and we monitored changes in the size and evolution of size aggregates over 600 s (Figure 4). Upon the introduction of the salt, both SeCobB_S_ and SeCobB_L_ exhibited two types of size distributions in the solution state (Eq. 5), designated as A and B in Figure 4. For both SeCobB_S_ and SeCobB_L_, within distribution 1, the hydrodynamic radius remained relatively stable at approximately 30 nm throughout the experiment. In contrast, within distribution 2, the hydrodynamic radius showed a remarkable increase from 90 to 300 nm over the observed time frame for SeCobB_L_. Conversely, for SeCobB_S_, within distribution 2, the variation in the hydrodynamic radius was much narrower, spanning from 35 to 50 nm. This substantial change in the hydrodynamic radius for SeCobB_L_ within distribution 2 suggests the progressive formation of larger protein complexes. This phenomenon is likely attributed to the strong interaction between the protein molecules and ZnCl_2_. Importantly, this observation strongly supports the findings of the experimental studies in fluorescence spectroscopy, which indicated the presence of more available zinc-binding sites within SeCobB_L_ than within SeCobB_S_. The average scattering intensity is known to be proportional to the size distribution of proteins in the solution. Therefore, we investigated its time-dependent variation immediately after the induction with ZnCl_2_. Figure 4C shows the average intensity as a function of time, denoted as I_t_ (normalized with the intensity at time zero), for SeCobB_S_ and SeCobB_L_ following ZnCl_2_ induction. It is evident from the plot that the average value of I_t_/I_o_ for SeCobB_S_ is lower than that for SeCobB_L_ and remains relatively constant throughout the observed time period. In contrast, for SeCobB_L_, the average intensity experiences a rapid increase immediately after induction, peaking at 50 s, after which the rate of increase in intensity slows down with time. The higher average scattering intensity and the rapid initial increase observed in SeCobB_L_, compared to SeCobB_S_, are consistent with the presence of a larger size variation within SeCobB_L_.

### 3.6 SeCobB–Zn^2+^ interaction studies by fluorescence spectroscopy

**FIGURE 5:**
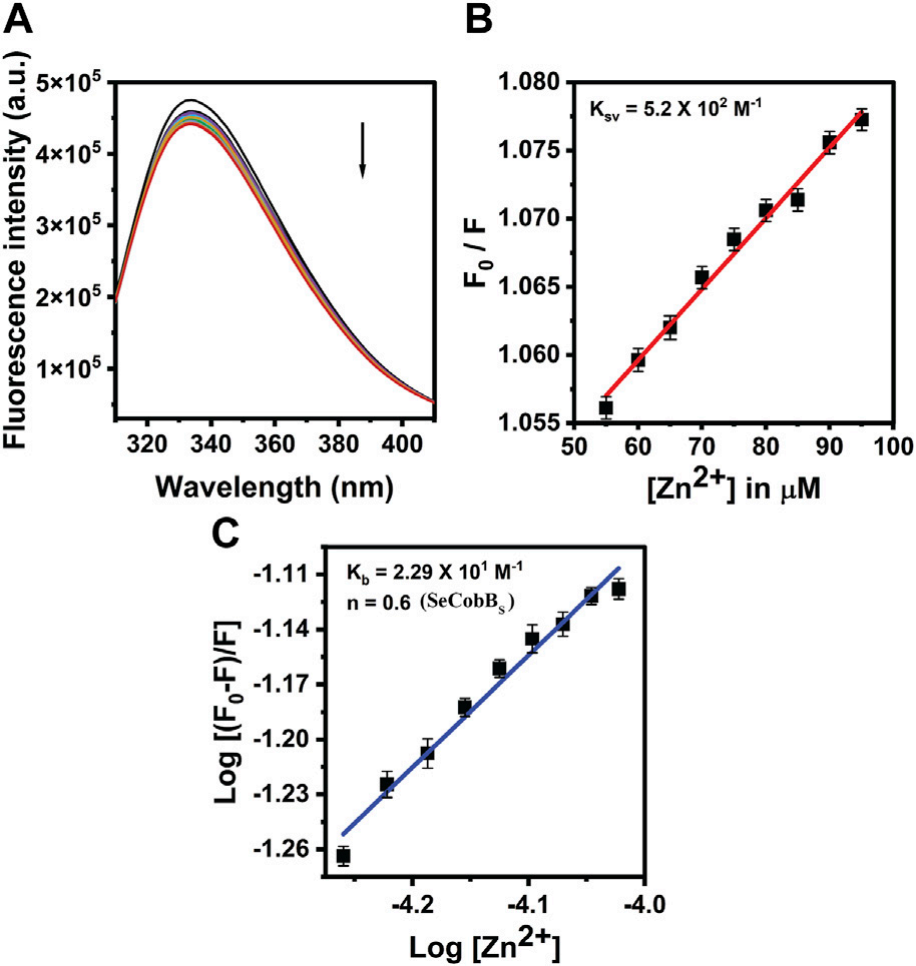
Fluorescence quenching studies of SeCobB_S_. **(A)** Intrinsic tryptophan fluorescence spectra of SeCobB_S_ (5 µM) in the presence of zinc ions (0–100 μM, at the rate of change of 5 µM per addition). Tryptophan fluorescence spectra were recorded in the range of 310–400 nm at 25°C. The excitation wavelength was 295 nm. The arrow indicates the effect of increasing concentration of Zn^2+^ on the tryptophan fluorescence emission of SeCobB_S_. **(B)** The linear fit of F_0_/F vs. [Zn^2+^] and Stern–Volmer quenching constant (K_SV_) was calculated using Eq. 3. **(C)** The plot represents the linear fit of log [(F0−F)/F] vs. log [Zn^2+^] for zinc ions, and the binding constant (K_b_) was estimated using Eq. 4. The experiment was performed in three biological replicates.

**FIGURE 6:**
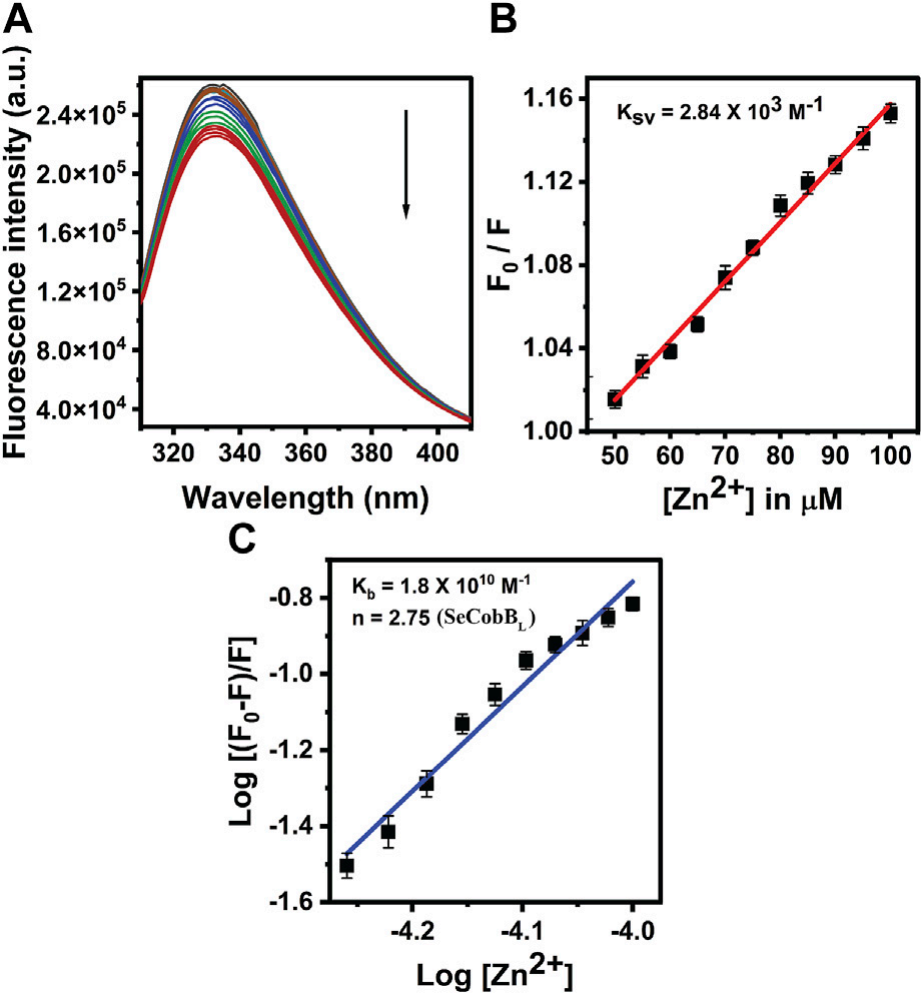
Fluorescence quenching studies of Zn^2+^ with SeCobB_L_. **(A)** Intrinsic tryptophan fluorescence spectra of SeCobB_L_ (5 µM) in the presence of zinc ions (0–100 μM, at the rate of change of 5 µM per addition). Tryptophan fluorescence spectra were recorded in the range of 310–400 nm at 25°C. The excitation wavelength was 295 nm. The arrow indicates the effect of increasing concentration of Zn^2+^ on the tryptophan fluorescence emission of SeCobB_L_. **(B)** The linear fit of F0/F vs. [Zn^2+^] and Stern–Volmer quenching constant (K_SV_) was calculated using Eq. 3. **(C)** The plot represents the linear fit of log [(F_0_−F)/F] vs. log [Zn^2+^] for zinc ions, and the binding constant (K_b_) was estimated using Eq. 4. The experiment was performed in three biological replicates.

The proteins containing an intrinsic fluorophore tryptophan can be used to monitor the binding of metal ions (Mattocks et al., 2021). The binding of Zn^2+^ ion with SeCobB_S_ and SeCobB_L_ was monitored by the quenching of tryptophan fluorescence upon the addition of zinc ions. A measure of 5 µM of SeCobB_S_ and SeCobB_L_ was titrated with zinc ions from 0 to 100 µM. Quenching of fluorescence intensity was observed upon the addition of Zn^2+^, which is suggestive of the interaction between Zn^2+^ and both protein isoforms (Figures 5A, 6A). It is observed from Figures 5A, 6A that the quenching in SeCobB_S_ is ∼6%, while that in SeCobB_L_ is ∼14%. This shows that zinc interacts strongly with SeCobB_L_ compared to SeCobB_S_.

The intrinsic fluorescence intensity of both protein isoforms decreased gradually upon increasing the concentration of zinc ions, but the saturation due to zinc quenching was observed earlier in SeCobB_S_ than that in SeCobB_L_ (Figures 5A, 6A). Stern–Volmer quenching (Eq. 6) and Scatchard analysis (Eq. 7) revealed that the binding affinity of Zn^2+^ with SeCobB_L_ is more than that with SeCobB_S_ (Table 1). The Stern–Volmer quenching constant for Zn^2+^ with SeCobB_S_ and SeCobB_L_ is 5.20 × 10^2^ M^−1^ and 2.84 × 10^3^ M^−1^, respectively (Figure 5B, 6B and Table 1). The extent of fluorescence quenching signifies the association of zinc ions with both protein isoforms. The state of equilibrium between free and bound proteins upon binding with small molecules is defined by the Scatchard equation.

**TABLE 1:** Stern–Volmer (K_sv_) and binding constant (K_b_) values of quenching using intrinsic tryptophan fluorescence between Zn^2+^ and SeCobB.

Protein	Binding data at 25°C (0–100 μM)		
	K_sv_	K_b_	n
**SeCobB**_**L**_ **-R**	2.84 × 10^3^	1.8 × 10^10^	2.75
**SeCobB**_**S**_ **-R**	5.20 × 10^2^	2.29 × 10	0.60

The association binding constant (K_b_) for SeCobB_S_ and SeCobB_L_ with zinc is 2.29 × 10 M^−1^ and 1.8 × 10^10^ M^−1^, with the binding stoichiometry/site (n) 0.60 and 2.75, respectively (Figures 5C, 6C and Table 1). This shows that the tendency of Zn^2+^ to bind with SeCobB_L_ is much higher than that with SeCobB_S_, which is also reflected in the number of binding sites. The *in vitro* deacetylase assay demonstrates that there is maximum inhibition in the deacetylase activity of SeCobB_S_ and SeCobB_L_ at 0.75 and 1 mM ZnCl_2,_ respectively. Beyond this concentration, saturation is attained in the band intensity. According to the relative intensity graph, 1.98% and 2.79% residual deacetylase activity of SeCobB_S_ and SeCobB_L_ was observed in the presence of 0.75 mM ZnCl_2_ compared to the activity in the absence of ZnCl_2_ (Figures 3A, B and Supplementary Figure S4). This result also corroborates with the fact that the number of zinc-binding sites in SeCobB_S_ is less than that in SeCobB_L._

**FIGURE 7:**
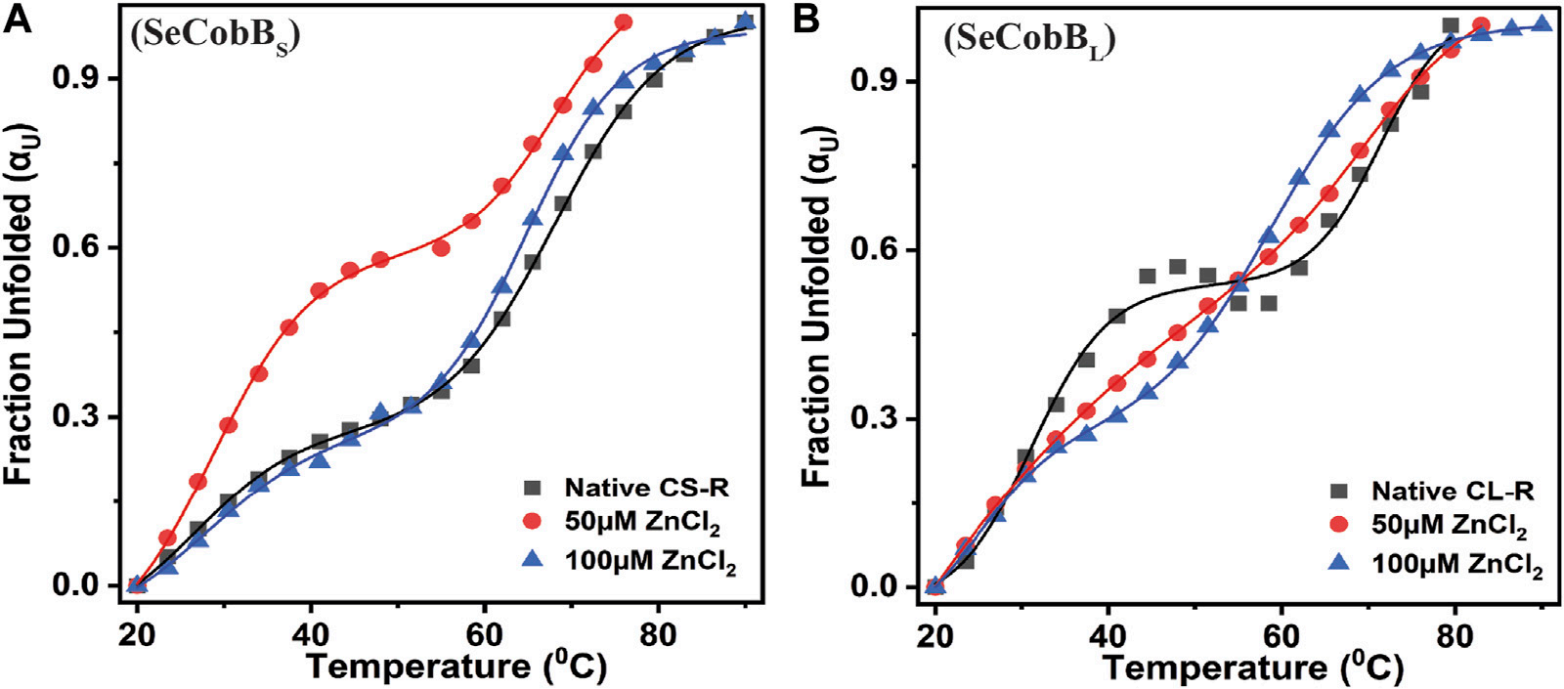
Effect of Zn^2+^ on the thermal stability of SeCobB_S_ **(A)** and SeCobB_L_ **(B)**. Thermal unfolding profiles for 5 µM SeCobB_S_ and SeCobB_L_ in the absence or presence of zinc ions (0–100 µM) in protein elution buffer (pH 8.0). Temperature-induced changes in the fraction of the unfolded state (α_U_) for SeCobB_S_ and SeCobB_L_ proteins. The profile has been normalized to a scale of 0–1. Symbols represent the experimental data points, and the solid lines represent the best fit according to the bi-dose curve fitting. The experiment was performed in three biological replicates.

The structural stability and integrity of a protein are essential to exhibit its function. Therefore, to assess the stability of SeCobB_S_ and SeCobB_L_, thermal denaturation experiments were conducted. The thermal denaturation of SeCobB_S_ and SeCobB_L_ carried out in the absence of zinc ions showed a bi-dose response model fitting, which revealed two thermal melting (T_m_) values for both protein isoforms (Figure 7). The T_m_ values for SeCobB_L_ are 31.20°C and 71.32°C, and those for SeCobB_S_ are 28.67°C and 67.94°C (Table 2) in the absence of zinc ions (Figure 7) (Table 2 and Figures 7A, B).

**TABLE 2:** Mid-point transition or T_m_, van’t Hoff enthalpy (ΔH_vH_), and entropy (ΔS) values associated with the thermal denaturation of SeCobB_L_ and SeCobB_S_ at different zinc ion concentrations.

Protein	Thermal stability (T_m_)								
ZnCl_2_ (µM)	0			50			100		
	T_m_	ΔH_VH_	ΔS	T_m_	ΔH_VH_	ΔS	T_m_	ΔH_VH_	ΔS
**SeCobB**_**L**_ **-R**	31.20	89.4	283.9	30.89	61.04	189.6	24.72	41.1	124.2
	71.32	110.8	333.4	71.12	105.9	320.8	58.91	100.6	406.7
**SeCobB**_**S**_ **-R**	28.67	54.4	164.3	26.62	60.1	214	27.75	58.6	176.4
	67.94	122.7	350.5	68.06	127.2	387.4	64.85	126.3	408.13
	**T**_**m**_ **in °C, ΔH_vH_ in kJ.mol** ^**−**^ **^1^,** **and ΔS in J.K** ^**−**^ **^1^ ** **mol** ^**−**^ **^1^**								

A similar bi-dose response fitting was also reported by Anand et al. (2019) for the Eis protein from *M. tuberculosis* and *M. smegmatis*. This shows that both protein isoforms have a sequential thermal denaturation mechanism. The thermal denaturation data clearly show that SeCobB_L_ is thermally more stable than SeCobB_S_ at ∼ 3°C–5°C. In the presence of Zn^2+^, the thermal stability of both SeCobB_S_ and SeCobB_L_ decreases but more significantly for SeCobB_L_. The addition of 100 µM Zn^2+^ reduces the T_m1_ value of SeCobB_S_ from 28.67°C to 27.75°C (ΔT_m1_ = 0.92°C) and the T_m2_ value of SeCobB_S_ from 67.94°C to 64.85°C (ΔT_m2_ = 3.09°C). In SeCobB_L_, an increase in the zinc ion concentration decreases the T_m1_ value from 31.20°C to 24.72°C (ΔT_m1_ = 6.48°C) and the T_m2_ value from 71.32°C to 58.91°C (ΔT_m2_ = 12.41°C), respectively. The ΔT_m1_ (0.92°C and 6.48°C for SeCobB_S_ and SeCobB_L_, respectively) and ΔT_m2_ values (3.09°C and 12.41°C for SeCobB_S_ and SeCobB_L_, respectively) in the presence of 100 µM ZnCl_2_ revealed that the interaction or binding of zinc ions decreased the thermal stability of SeCobB_L_ to a greater extent than that of SeCobB_S_.

The higher decrease in the thermal stability of the former in the presence of zinc ions may be due to the greater binding of the zinc ions with SeCobB_L_ than with SeCobB_S_. The calculation of the van’t Hoff enthalpy of the thermal denaturation curves revealed that the van’t Hoff enthalpy for SeCobB_L_ for the first thermal melting transition decreased significantly from 89.4 to 41.1 kJ mol^−1^ (Table 2), while for the second thermal melting transition, it decreased from 110.8 to 100.6 kJ mol^−1^ (Table 2 and Figure 7) upon the addition of zinc ions. The greater decrease in the van’t Hoff enthalpy for the first denaturation transition may be due to the perturbation in the oligomeric assembly of SeCobB_L_ upon the addition of zinc ions. Since SeCobB_S_ is monomeric in nature, the change in van’t Hoff enthalpy upon the addition of zinc ions is not very significant.

## 4 Discussion

### 4.1 SeCobB is an NAD^+^-dependent lysine histone deacetylase

Prokaryotic NAD^+^-dependent deacetylases, predominantly known as CobB, the mammalian SIRT5 isoforms, are fairly conserved in Gram-negative bacteria and have been extensively investigated in *S. enterica* and *E. coli* (Mishra et al., 2022). Gram-negative species like *Vibrio*, *Mycobacterium*, *Shigella*, and *Klebsiella* are of clinical importance due to their ability to cause infection in the host. The sirtuin core domain in SeCobB is evolutionarily more conserved in archaea than in Eukarya (Buck et al., 2004). Numerous biological processes including cellular metabolism, transcriptional repression, and epigenetic alterations are influenced by the sirtuin-mediated regulation of acetylation. The deacetylase protein, frequently mentioned as CobB in the majority of the bacterial operations, has profound relevance in *Salmonella* physiology, like survival under stress conditions and regulation of virulence (Li et al., 2010; Liu et al., 2018). Escalante-Semerena (2010) reported predominant deacetylase activity of SeCobB_S_ over SeCobB_L_ in removing the lysine residue (K609) from acetylated acetyl CoA synthase (Acs^Ac^). Our data are the first report to biochemically prove SeCobB to be a NAD^+^-dependent histone lysine deacetylase (Figure 2), justifying them to be a class III deacetylase. We found the complete absence of acetylated H3 and H4 bands at 200 ng of SeCobB_S_ (Figure 2A), which are still visible in the case of SeCobB_L_ (Figure 2B). The higher deacetylase activity of SeCobB_S_ than that of SeCobB_L_ is consistent with an earlier report. Only two such previous reports demonstrated eukaryotic histones as a substrate for a bacterial deacetylase (Zhao et al., 2004; Mishra et al., 2022). *In vitro* deacetylation of core histones by SeCobB clearly indicates the ability of a bacterial deacetylase to alter host proteins during an infection.

### 4.2 Structure prediction and molecular docking studies

Zinc metalloenzymes are therapeutic targets in cancer, cardiac disease, bacterial infection, and Alzheimer’s disease. The majority of these enzymes are targeted by a potential drug candidate by investigating the interaction with the Zn^2+^ bound to them. As a result, the precise prediction of the protein–Zn^2+^ interaction is a key part of computational docking and virtual screening against Zn^2+^-binding proteins.

The Ramachandran plot was used to define the best modeled structure of SeCobB_S_ based on the C-score, and software applications like PROCHECK, ERRAT, and Verify3D were used for further refinement (Supplementary Figure S4). The polished ERRAT server output of the SeCobB structure is shown as a function of error values *versus* amino acids in Supplementary Figure S4. With a resolution of 2–3 Å, the protein structure received a score >90%. The red and yellow sections of the ERRAT graph reflect uncertain parts of the structure, while the white portions show the definite parts. This plot analysis identifies residues with error values >95% and 99% in very less time (Tables 3, 4).

**TABLE 3:** Qualitative analysis for SeCobB_S_ after refinement through different webservers.

SAVES meta-server result (PDB file)	
PROCHECK	Number of allowed residues: 191 (90.1%); number of disallowed residues: 2 (0.9%)
Verify3D	3D/1D profile of residues (scored by 80% of amino acids): 74.15% (>=0.2)
ERRAT	Quality factor: 93.1818
ProSA	Z-score: −7.05

**TABLE 4:** Qualitative analysis for SeCobB_L_ after refinement through different webservers.

SAVES meta-server result (PDB file)	
PROCHECK	Number of allowed residues: 212 (89.8%); number of disallowed residues: 4 (1.7%)
Verify3D	3D/1D profile of residues (scored by 80% of amino acids): 72.53% (>=0.2)
ERRAT	Quality factor: 94.024
ProSA	Z-score: −7.92

Predictability of an anticipated model is determined using the ProSA webserver. It reveals the accuracy of the modeled protein with respect to the experimentally crystallized structure. It also generates a Z-score that represents the overall model quality. Its value is reflected in a plot of all experimentally determined protein chains in the current PDB. Different colors differentiate groupings of structures from different sources (X-ray and NMR) in this figure.

The software application used here to determine the amino acid residues involved in the SeCobB–Zn^2+^ interaction follows different algorithms and force fields issued for docking purposes. The residues that most often bind to metal ions are CYS, HIS, GLU, and ASP (Auld, 2001; Golovin et al., 2005) because the atoms of their charged side chains can coordinate with metal ions. We performed site-specific docking using AutoDock, in which Cys 118 and Cys 137 were defined in a grid box to facilitate the specific interaction of Zn^2+^ toward these two predicted sites for SeCobB_S_. The same exercise was also performed for SeCobB_L._ Cys 118 and Cys 137 are annotated as Cys 155 and Cys 174 in the larger isoform. However, no such specific Zn^2+^-binding sites were defined in the case of blind docking; instead, the designed server had specified the grid box based on their template structure. Since the parameters used for both the techniques are different, Zn^2+^ might have more binding affinity toward one cysteine than the other. Further experiments are required to ascertain the binding affinity of Zn^2+^ toward either of the cysteine residues.

### 4.3 Zn^2+^ inhibits the *in vitro* deacetylase activity of SeCobB

NAD^+^-dependent protein deacetylase activity is critical to the physiological function of proteins belonging to the SIR2 family (Imai et al., 2000; Landry et al., 2000; Smith et al., 2000). Zinc has two major functions; either it can serve as a cofactor of enzyme catalysis, or it can act as an inhibitor of enzyme function. Zinc-mediated inhibition of enzyme activity can occur through three different mechanisms, namely, direct binding to the enzyme active site, allosteric mode of inhibition, and inhibition induced by binding to Zn^2+^ consecutive to catalytic zinc. The binding affinity of Zn^2+^ toward a protein, which is necessary to establish the physiological significance of zinc-mediated inhibition, varies from micromolar to picomolar concentration. Zinc-mediated inhibition of the enzyme phosphoglucomutase has been reported, which uses magnesium as its cofactor. The concentration of Zn^2+^ required for its inhibition is > 32 pmol/L (Magneson et al., 1987). The Zn^2+^ concentration beyond 1 mM is cytotoxic to the cells (McDevitt et al., 2011), hence determining the pathophysiological concentration. We performed *in vitro* histone deacetylase assay using 100 ng of purified SeCobB_S_ and SeCobB_L_ separately with various concentrations of ZnCl_2_ (0.25–1.25 mM). With the increasing concentration of ZnCl_2_, there was a sequential inhibition in the catalytic activity of both isoforms, which was restored in the absence of ZnCl_2_ (Figures 3A, B). The inhibition and restoration of deacetylase activity were ascertained by the enhanced and diminished band intensity captured from chemiluminescence data. This manifested the role of Zn^2+^ ions in inhibiting the catalytic activity of both isoforms. However, the extent of inhibition in the biochemical activity was different for both. The maximum inhibition of SeCobB_S_ activity was achieved with 1.25 mM ZnCl_2_, whereas for SeCobB_L,_ it was at 1 mM (Supplementary Figure S6).

### 4.4 Fluorescence spectroscopy studies

Interesting insights into the importance of Zn^2+^ in the field of nutrition, enzyme catalysis, protein biochemistry, and cellular biology have been obtained with the development of analytical techniques, like fluorescence spectroscopy, with a high sensitive level of detection (Maret et al., 2001; Maret, 2013b). This has shed light on the zinc-mediated alteration in the structure–function regulation of enzymes involved in various cellular processes. Since SeCob_B_ contains three tryptophan residues, we used fluorescence spectroscopy to record any changes in the thermal and structural stability of the protein due to the Zn^2+^–SeCobB interaction. A quenching study was done to define the binding affinity of Zn^2+^ with both SeCobB_S_ and SeCobB_L_. Data obtained from fluorescence quenching studies displayed a reasonable interaction between Zn^2+^ and SeCobB. The K_sv_ value for SeCobB_S_ is 10 times lower than that of SeCobB_L_ (Table 1), suggesting the requirement of a comparatively higher ZnCl_2_ concentration for interaction equivalent to SeCobB_L_. The binding constant (K_b_) for SeCobB_S_ is 10^9^ times less than that for SeCobB_L_, also supported by the number of Zn^2+^-binding sites (Table 1). The binding affinity of Zn^2+^ toward SeCobB_L_ is higher than that toward SeCobB_S_. SeCobB_L_ is an oligomer in solution, whereas SeCobB_S_ is a monomer (Figure 1C). Thus, being an oligomer, SeCobB_L_ confers a greater number of available zinc-binding sites, resulting in adequate binding and relevant interactions.

The thermal denaturation graph of both isoforms illustrates the biphasic melting curve both in the absence and presence of Zn^2+^ (Figure 7). This may be because of the independent melting of the NAD^+^-binding Rossmann fold domain and Zn^2+^-binding domain, which are the characteristic domains of the SeCobB predicted structure (Gao et al., 2020).

The initial 37-amino acid stretch in the N-terminus of SeCobB_L_ is 37% hydrophobic, which is a potential reason for its oligomerization. Moreover, DLS results demonstrate the narrow size distribution in SeCobB_L_ (Figure 1F) that facilitates more Zn^2+^-binding sites than in SeCobB_S_ (Figure 1E). However, we conducted more detailed DLS studies to understand the effect of Zn^2+^ on the structural properties of SeCobB and their dependence on temperature (Figure 4) in order to correlate with fluorescence spectroscopy studies. Induction with 250 µM ZnCl_2_ at 20°C led to an increase in the scattering intensity of both SeCobB_S_ and SeCobB_L_ within 50 s of salt addition. However, the time-dependent leap in the scattering intensity (I_t_) upon ZnCl_2_ addition was ∼9 times more in SeCobB_L_ than that in SeCobB_S_, implying that zinc mediated a profound impact on the larger isoform than on the shorter isoform.

## 5 Conclusion

This is the first study to experimentally report that the predicted zinc-binding motif of *S. enterica* CobB, present within the shorter isoform, SeCobB_S_ (38–273 amino acid position), has a low affinity for Zn^2+^, and we hypothesize that it may be true for other bacterial CobB proteins. The NTD of SeCobB_L_ helps in the formation of a stable oligomer, and it is the main site for the Zn^2+^–CobB interaction. This is supported by a ninefold increase in the scattering intensity of recombinant SeCobB_L_ on the addition of ZnCl_2_, whereas there was hardly any change in the scattering intensity of SeCobB_S_ (≤1.5-fold). Higher affinity toward SeCobB_L_ is also validated by the association constant (K_SV_) and binding constant (K_b_) values, which are 10 times and 10^9^ times more than that for SeCobB_S_, respectively_._ The definite number of zinc-binding sites in the larger isoform also corroborates to negligible binding with its truncated form, i.e., SeCobB_S._ Thermal stability of SeCobB_L_ was significantly reduced compared to that of SeCobB_S_ in the presence of Zn^2+^. Zn^2+^ inhibited histone deacetylase activity of SeCobB at a higher concentration. Taken together, Zn^2+^ induced structural changes, and inhibition of deacetylase activity of SeCobB delineates the function of the predicted zinc-binding motif of bacterial CobB. We further predict that the zinc-binding domain is not a suitable target for future drug development.

## Data Availability

The raw data supporting the conclusion of this article will be made available by the authors, without undue reservation.
