# Supplementary figures and images for: An insight into the role of the N-terminal domain of Salmonella CobB in oligomerization and Zn2+ mediated inhibition of the deacetylase activity
Source: Front Mol Biosci. 2024 Mar 13;11:1345158. doi: 10.3389/fmolb.2024.1345158 (PMC10965786; doi:10.3389/fmolb.2024.1345158)

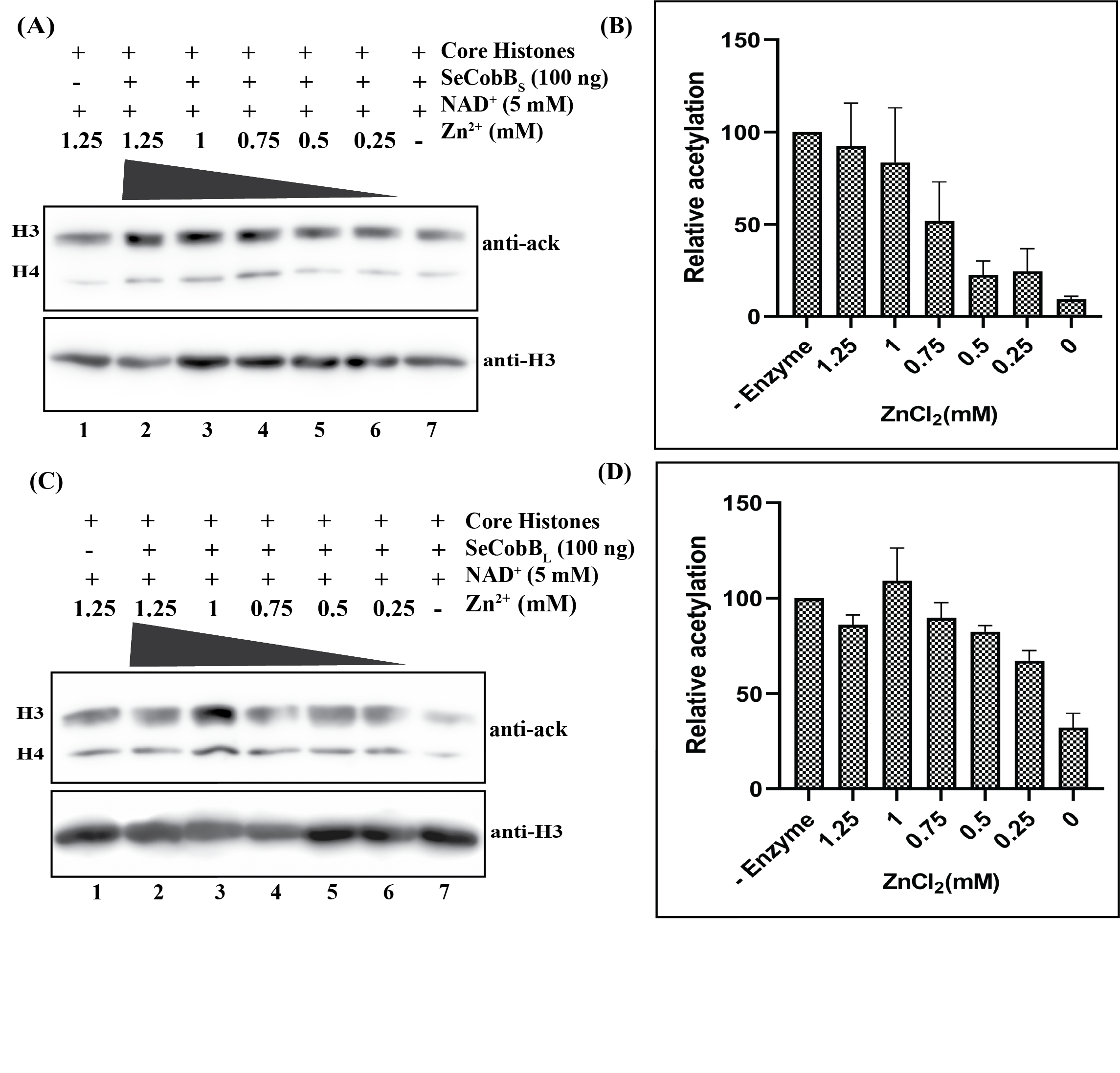

Supplement: Supplementary file 1 [file Image6.TIF]

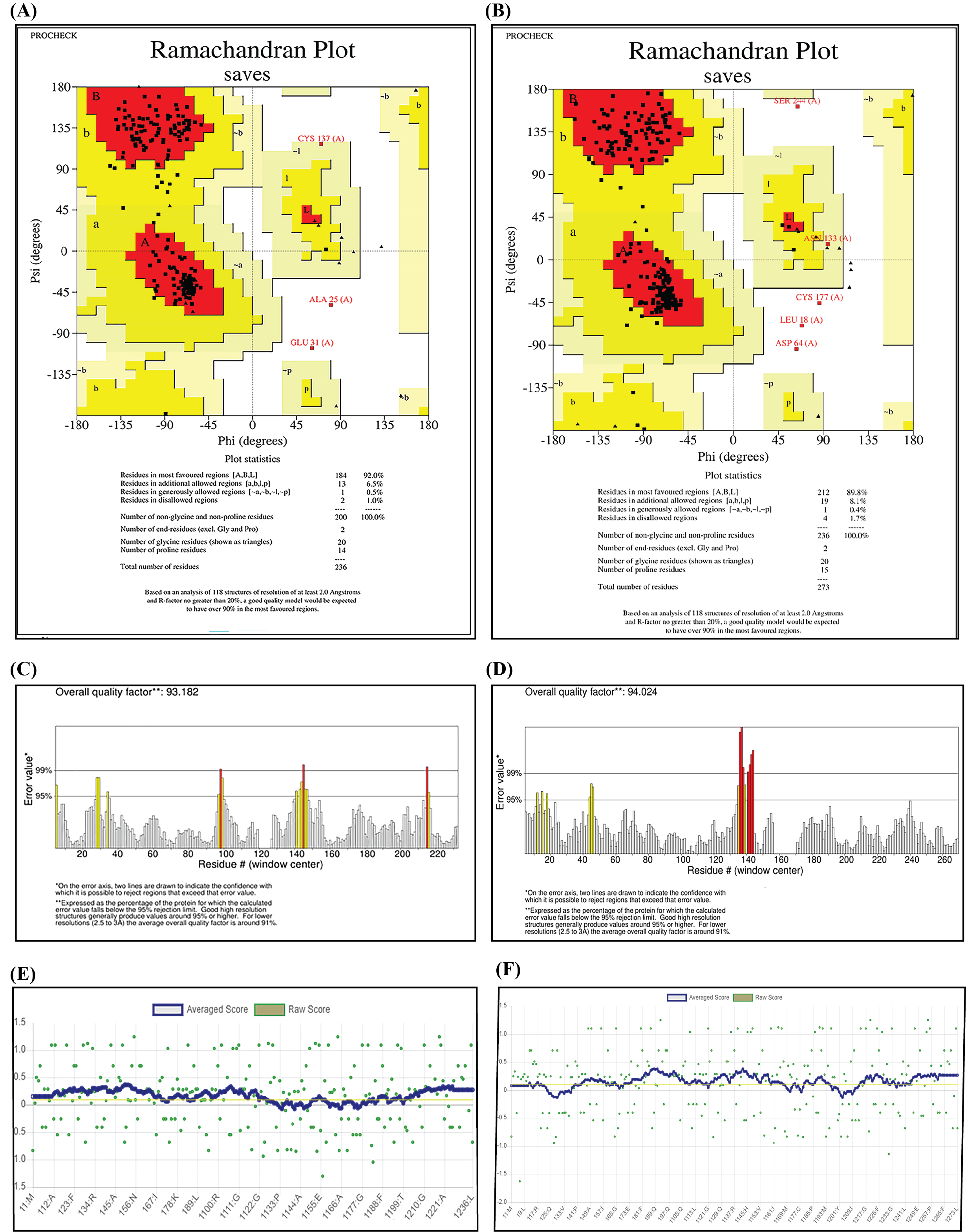

Supplement: Supplementary file 2 [file Image3.TIF]

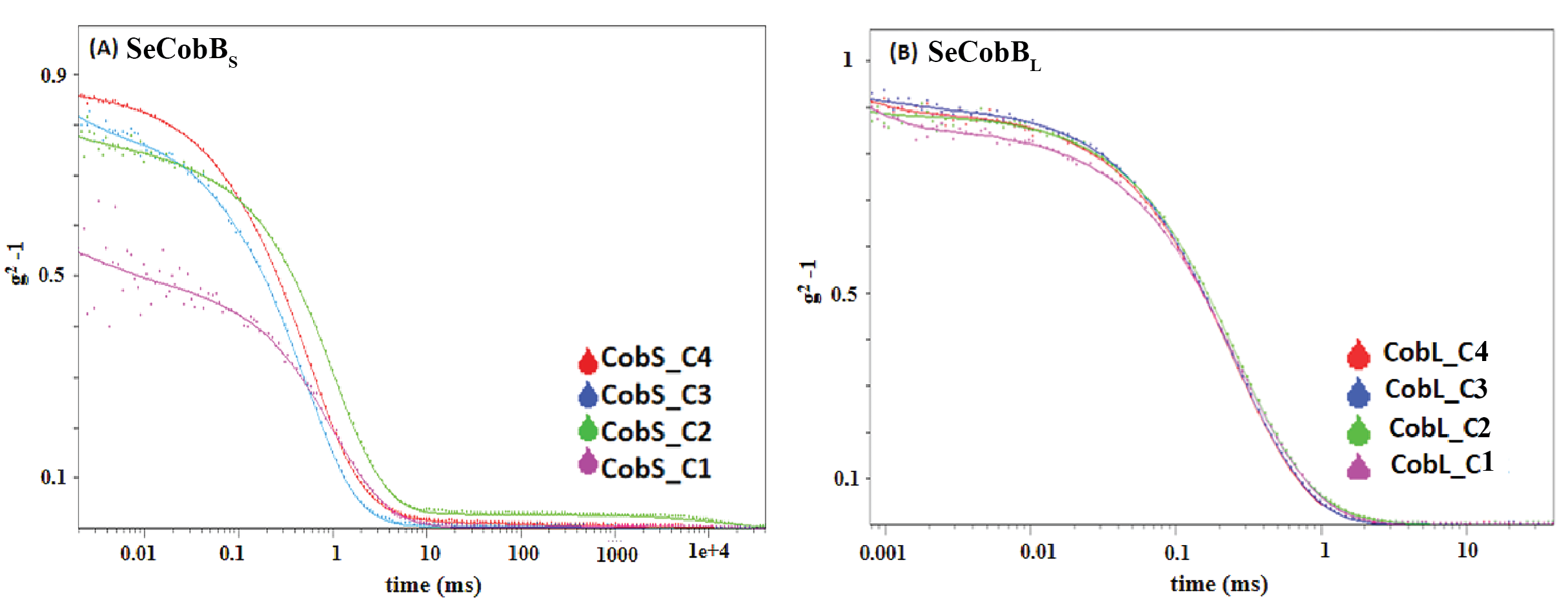

Supplement: Supplementary file 3 [file Image4.TIF]

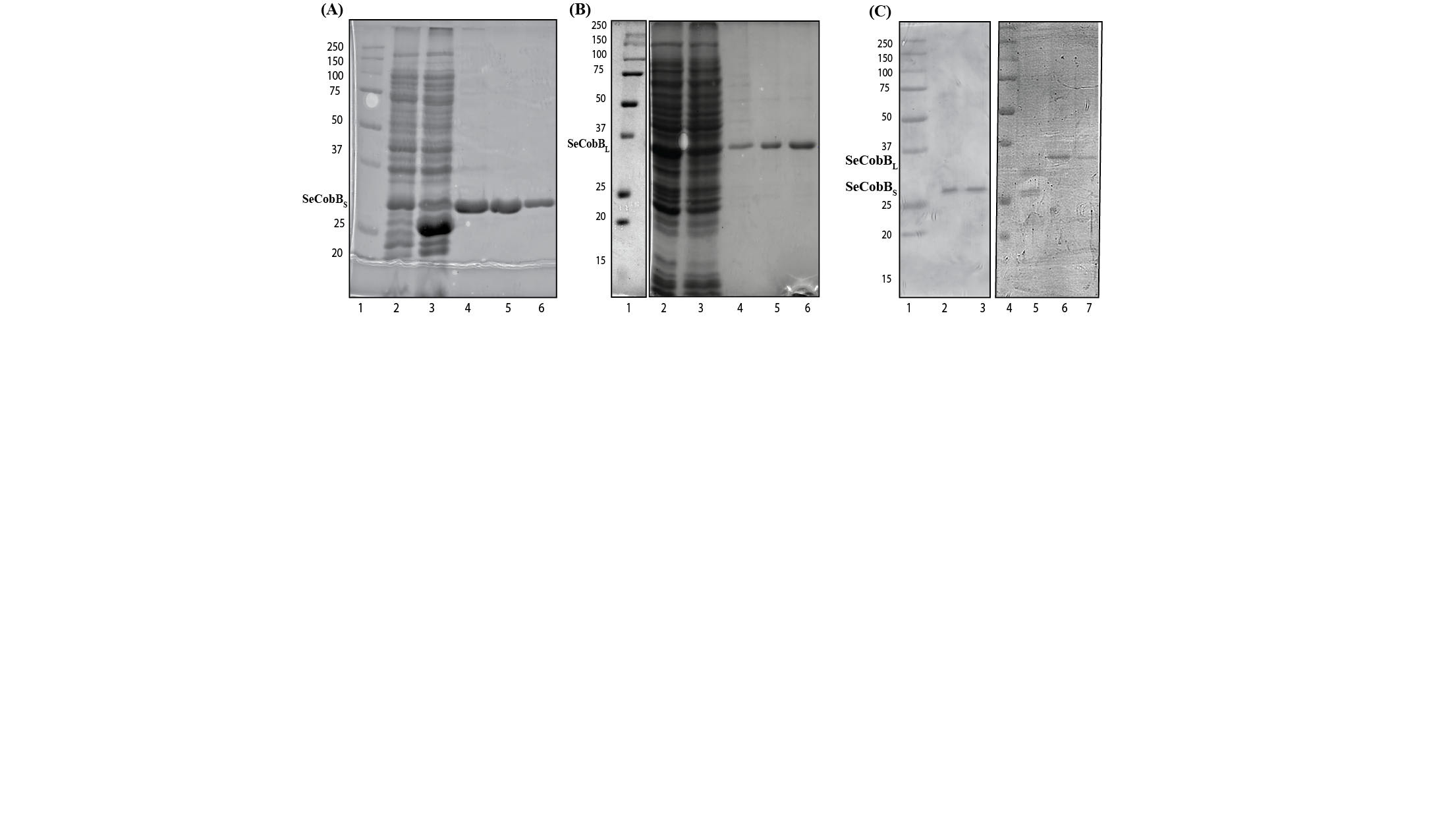

Supplement: Supplementary file 4 [file Image2.TIF]

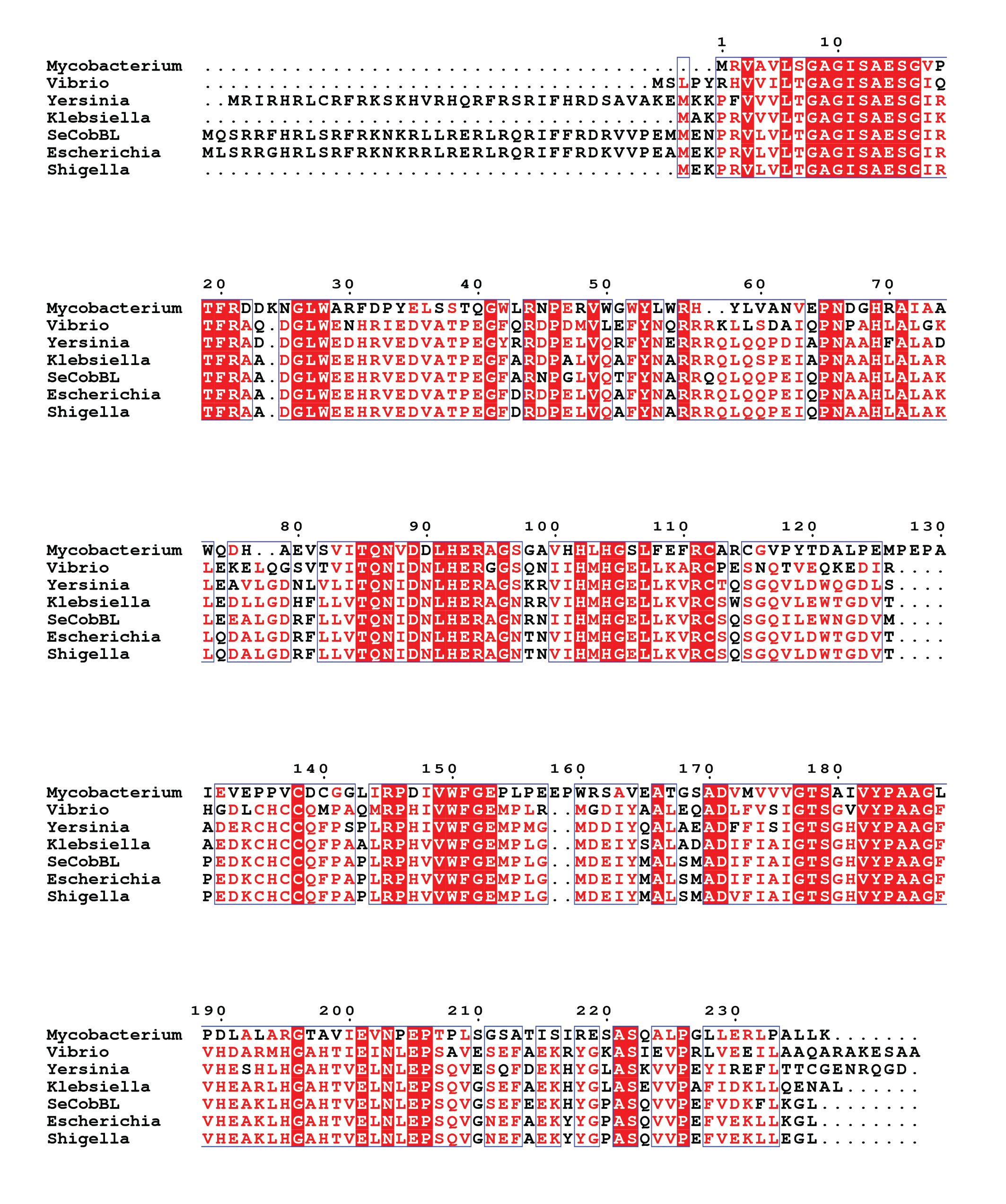

Supplement: Supplementary file 5 [file Image1.TIF]

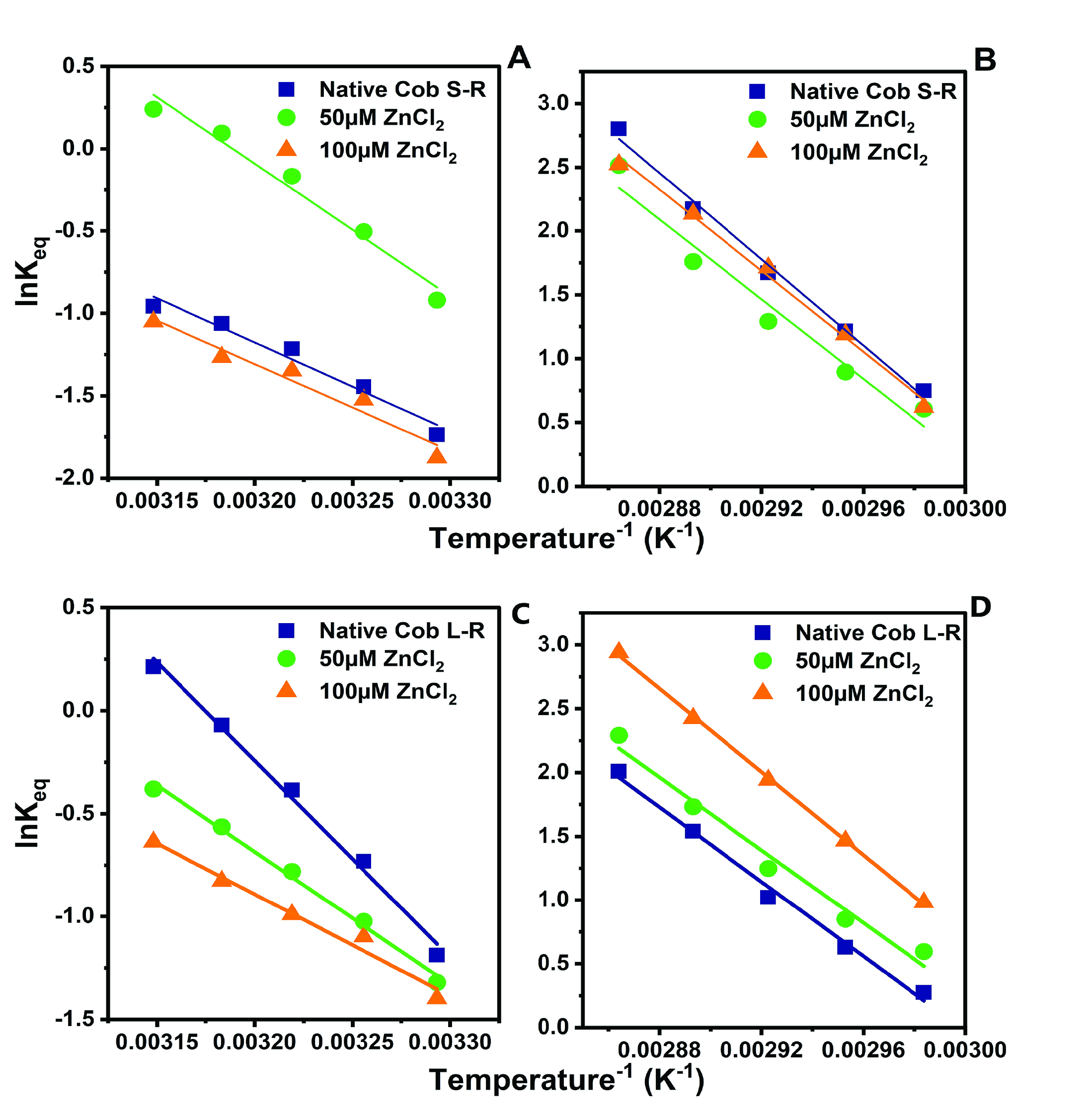

Supplement: Supplementary file 6 [file Image7.TIF]

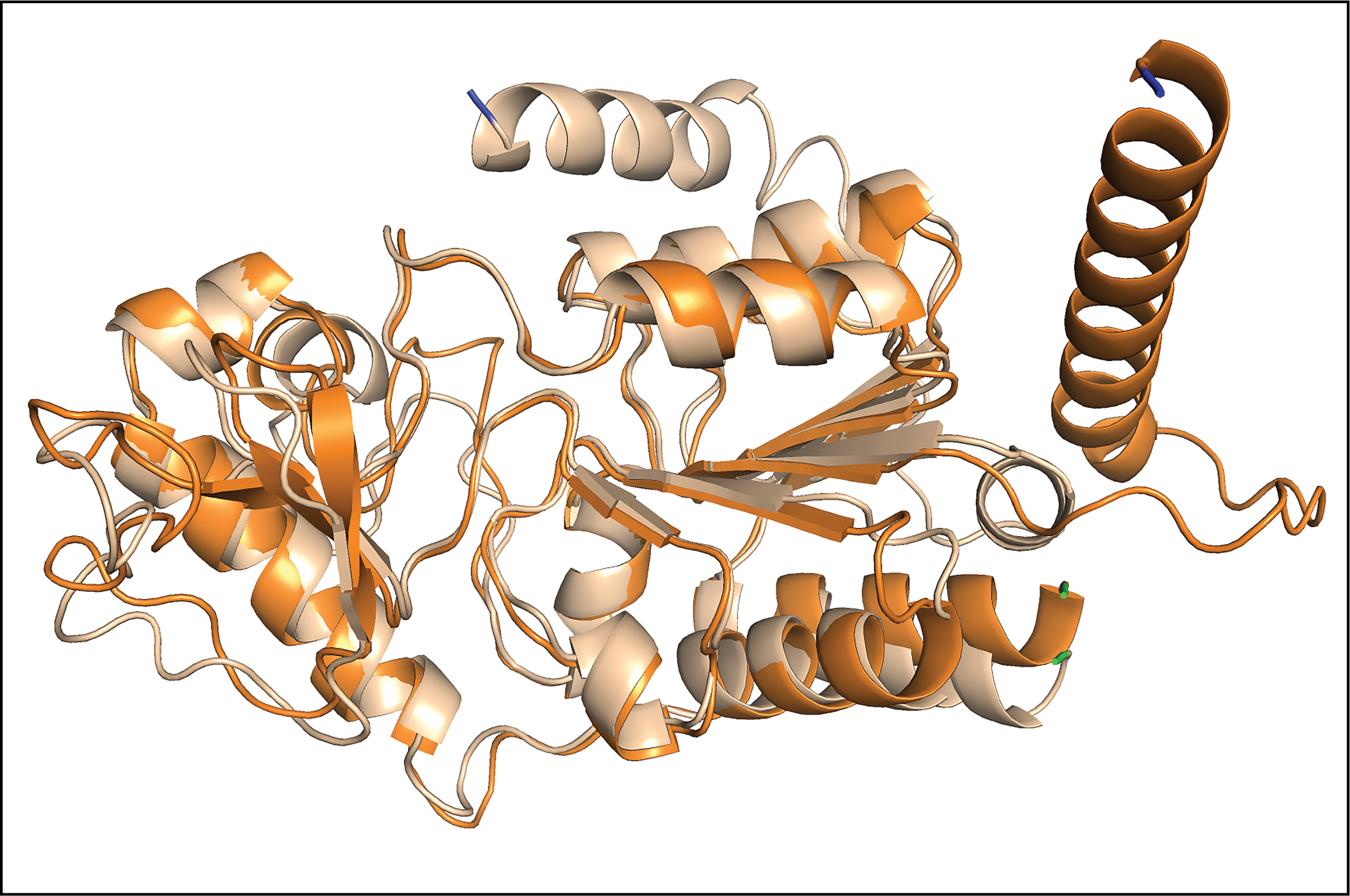

Supplement: Supplementary file 7 [file Image5.TIF]
