# Supplementary material for: An insight into the role of the N-terminal domain of Salmonella CobB in oligomerization and Zn2+ mediated inhibition of the deacetylase activity
Source: Front Mol Biosci. 2024 Mar 13;11:1345158. doi: 10.3389/fmolb.2024.1345158 (PMC10965786; doi:10.3389/fmolb.2024.1345158)
Supplement: Supplementary file 8 [file DataSheet1.docx]

**Supplementary information (File 1)**

**An insight into role of N-terminal domain of *Salmonella enterica* CobB in oligomerization and Zn^2+^mediated inhibition of deacetylase activity.**

Shibangini Beura^1^, Pulak Pritam^2^, AjitKumar Dhal^1^, Arindam Jana^1^, Aiswarya Dash^1^, Pritisundar Mohanty^3^, Alok Kumar Panda^2#^, and Rahul Modak^1^*

1 Infection and Epigenetics Laboratory, School of Biotechnology, Kalinga Institute of Industrial Technology (KIIT), Bhubaneswar, Odisha, India

2 Environmental Science Laboratory, School of Applied Sciences, Kalinga Institute of Industrial Technology (KIIT), Bhubaneswar, Odisha, India

3. School of Biotechnology, Kalinga Institute of Industrial Technology (KIIT), Bhubaneswar, Odisha, India

* To whom correspondence should be addressed. Tel: +91-9040442305, Email: [rahul.modak@kiitbiotech.ac.in](mailto:Rahul.modak@kiitbiotech.ac.in), [rahulmodak123@gmail.com](mailto:rahulmodak123@gmail.com)

# Co-corresponding author [alok.pandafch@kiit.ac.in](mailto:alok.pandafch@kiit.ac.in)

**Material and Methods**

**Bioinformatic analysis**

Sequence homology of SeCobB with NAD^+^ dependent deacetylases across enteropathogenic gram negative bacteria was performed using Clustal Omega (https://www.ebi.ac.uk/Tools/msa/clustalo/) multiple sequence alignment tool with post analysis from ESPript program. [[1](#_ENREF_1)]

**PCR and Restriction enzyme Cloning**

The genomic DNA of *Salmonella enterica* subspecies I serovar Enteritidis str. P125109 was used as the DNA template to clone *Se*CobBs (722 bp) and *Se*CobB_L_ (822 bp) respectively. *E. coli* DH5α strain was used as the cloning host. The PCR amplification of *Se*CobBs was performed in the Eppendorf Mastercycler gradient PCR thermal by using the high-fidelity Ex-prime Taq polymerase from *Genet*-bio. The primers for the respective amplifications are listed in Table-5. Accordingly, the following components were added to the 50μl PCR reaction: H_2_O ultrapure 39.5 μl, 10X-PCR Buffer -5 μl, DNTPs (10 mM) 2 μl, forward and reverse primer (10 pmole/μl) 1 μl, taq DNA polymerase (5 U/μl) 0.5 μl and DNA template (1 μl). A reaction mixture containing all the components except for the template DNA was used as negative control. The PCR profile consisted of 35 cycles including denaturing temperature for 30 s, annealing for 30 s and extension for 60 s, at 95°C, 60°C and 72°C, respectively. The PCR products were evaluated by electrophoresis on 1.0% agarose gel and visualised under UV light transilluminator. The amplicons and the plasmid DNA (pET-28a) were digested with BamHI and XhoI restriction enzymes. The digested amplicons and plasmid DNA were gel purified using the Genet-Bio gel purification kit. The purified amplicons were individually ligated to pET-28a at 16°C overnight. The ligated mixtures were independently transformed into *E. coli* DH5α chemical competent cells. The plate was incubated at 37^0^C for 12-15 hours. The transformed colonies were screened by PCR using gene specific/T7forward primer and gene-specific reverse primers. (Table 1)

**Table 1: Primers used in cloning of SeCobB**

| *Se*CobB_S_ F. P | 5’ CGC GGATCC ATGGAAAACCCAAGAGTATTAGTC 3’ |
| --- | --- |
| *Se*CobB_S_ R. P | 5’ CCG CTCGAG CTACAGCCCTTTCAGGAATTTATC 3’ |
| *Se* T7_R. P | CAAAAAACCCCTCAAGACCC |
| *Se*CobB_L_ F. P | CGGGATCC ATG CAG TCG CGT CGG TTT C |
| *Se*CobB_L_ R. P | CCG CTCGAG CTA CAG CCC TTT CAG GAA TTT AT |

**Purification using Ni^2+^- NTA affinity chromatography.**

Recombinant SeCobBs-pET28a and SeCobB_L_-Pet28a constructs were transformed into *E. coli* BL21 codon plus (DE3) for protein induction. Both the N-terminal 6X-His-tagged-SeCobB isoforms were expressed with 0.5mM IPTG induction at 37^0^C for 4h at 150 rpm. Cell pellets were resuspended in protein elution buffer (25mM Tris pH 8, 200mM NaCl, 2mM β-mercaptoethanol, 5% Glycerol). After sonication, the cell lysates were subjected to centrifugation at 12000g, 4^0^C and the supernatant containing the soluble fraction were set for binding with Ni^2+^NTA agarose resin (Qiagen). The unbound impurities were removed with subsequent imidazole washes. Finally, the proteins were eluted in 100mM imidazole and were subjected to buffer exchange on Hi Trap Desalting column (GE HealthCare) using AKTA Pure L (GE Life HealthCare). Collected eluates were resolved on SDS PAGE and were stained with CBB R-250 (Sigma) to know the purity of the samples.

**Results**


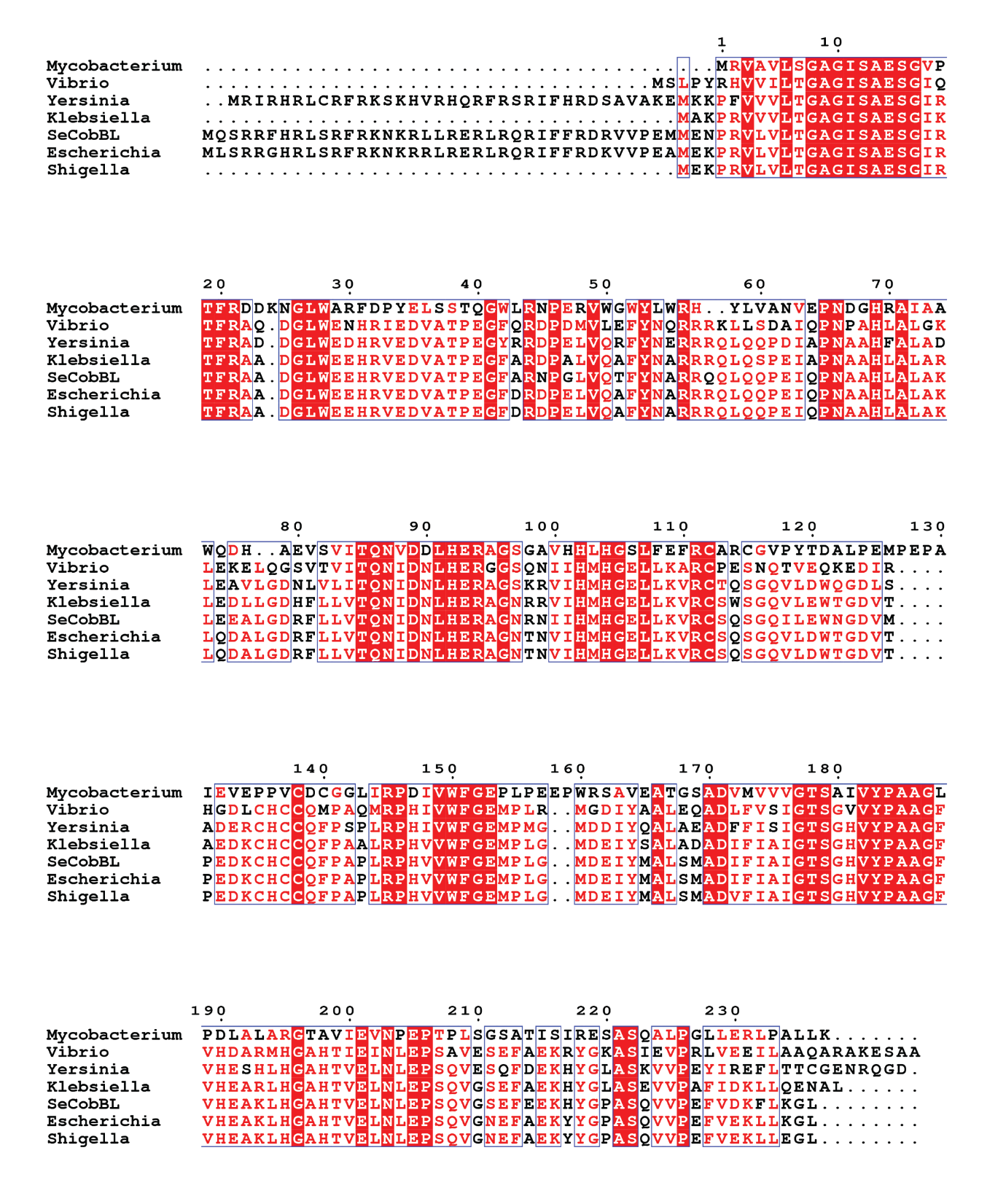


**Figure S1.** Multiple sequence alignment of SeCobB_L_ with other NAD^+^ dependent deacylase homologues present in gram negative bacteria. Sequences highlighted in red and black boxes denote most identical to similar residues, respectively.


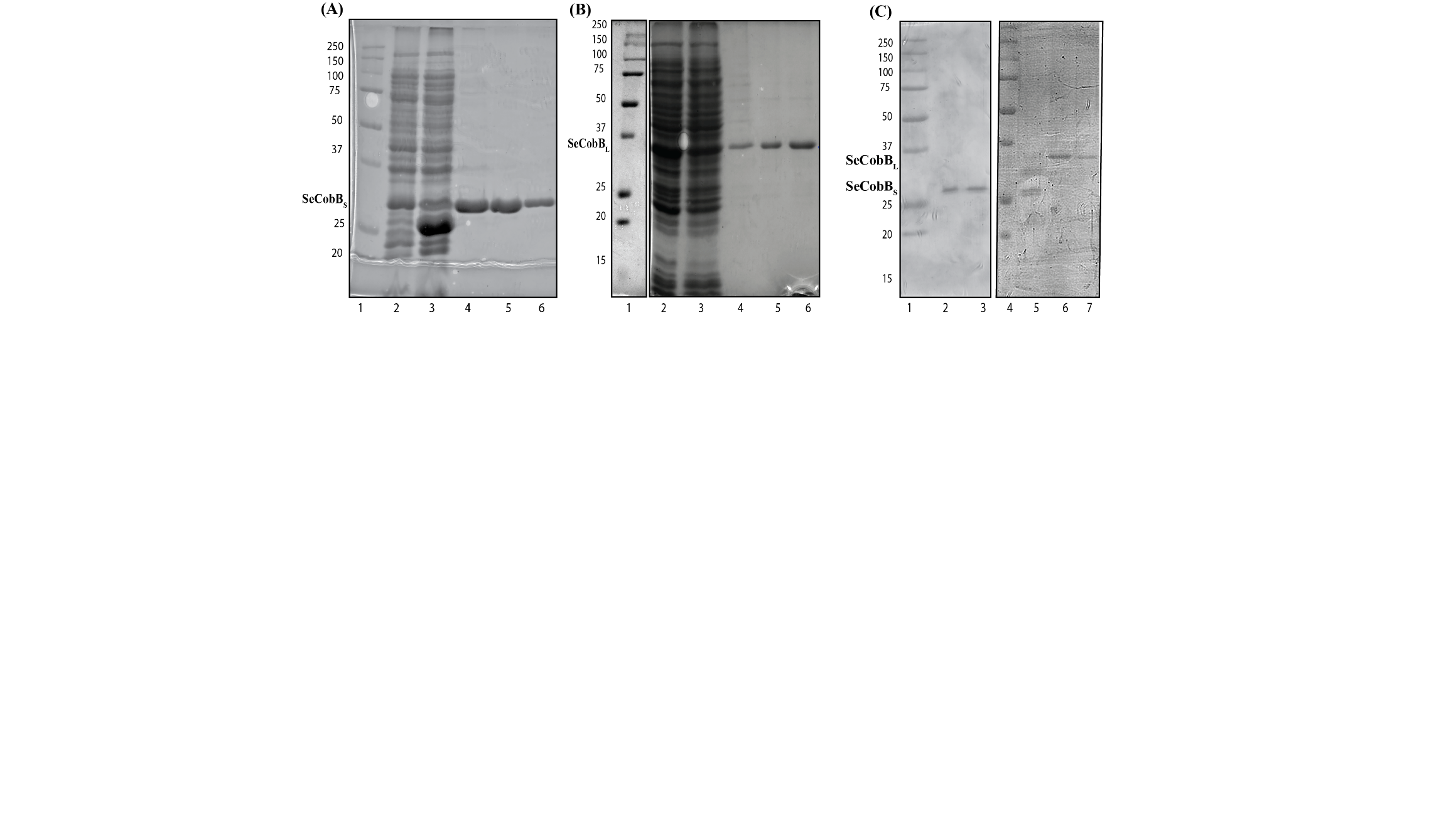


**Figure S2.** Purification profile of SeCobB_S_ (A) and SeCobB_L_ (B) using Ni^2+^-NTA chromatography and SDS-PAGE of their corresponding GPC eluates (C). Purified SeCobB_S_ and SeCobB_L_ bands were observed at 30.3 kDa and 34 kDa respectively. Lane 1(A)/(B) – Biorad protein Ladder, Lane 2(A)/(B)- Supernatant of the crude lysate, Lane 3(A)/(B)- Flowthrough after O/N binding with Ni^2+^-NTA resin. Lane 4-6 (A)/(B)- purified eluates at 200 mM imidazole. Lane 1,4(C)- Biorad Protein Ladder, Lane 2,3- GPC eluates of recombinant SeCobB_S_ at 30.3 kDa, Lane 5,6,7- GPC eluates of recombinant SeCobB_L_ at 34 kDa_._


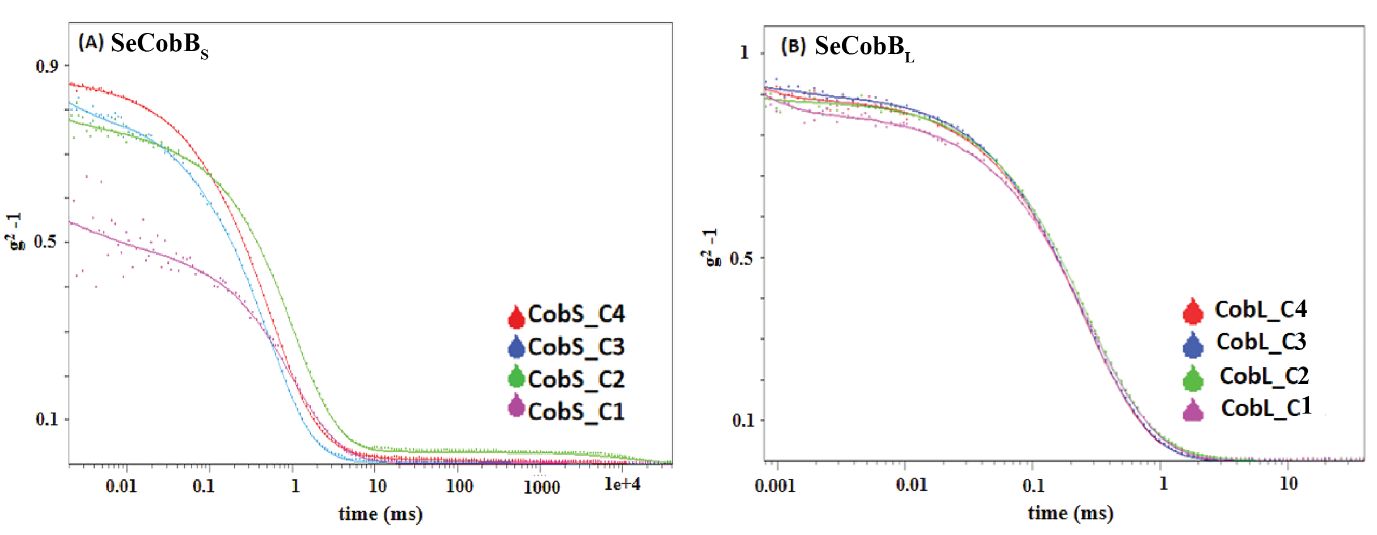


**Figure S3.**  Intensity-Intensity correlation function from DLS experiment (denoted as circle) are plotted as a function of time [[2](#_ENREF_2)] against different concentration of recombinant SeCobB_S_ (A) and SeCobB_L_ (B). The concentration CobS_C1 to CobS_C4 correspond to 93 ug/ml, 187 ug/ml, 375 ug/ml, and 750 ug/ml respectively and the concentration CobL_C1 to CobL_C4 correspond to 100 ug/ml, 200 ug/ml, 500ug/ml, and 800ug/ml respectively. The lines are the fit with the CONTIN based method.


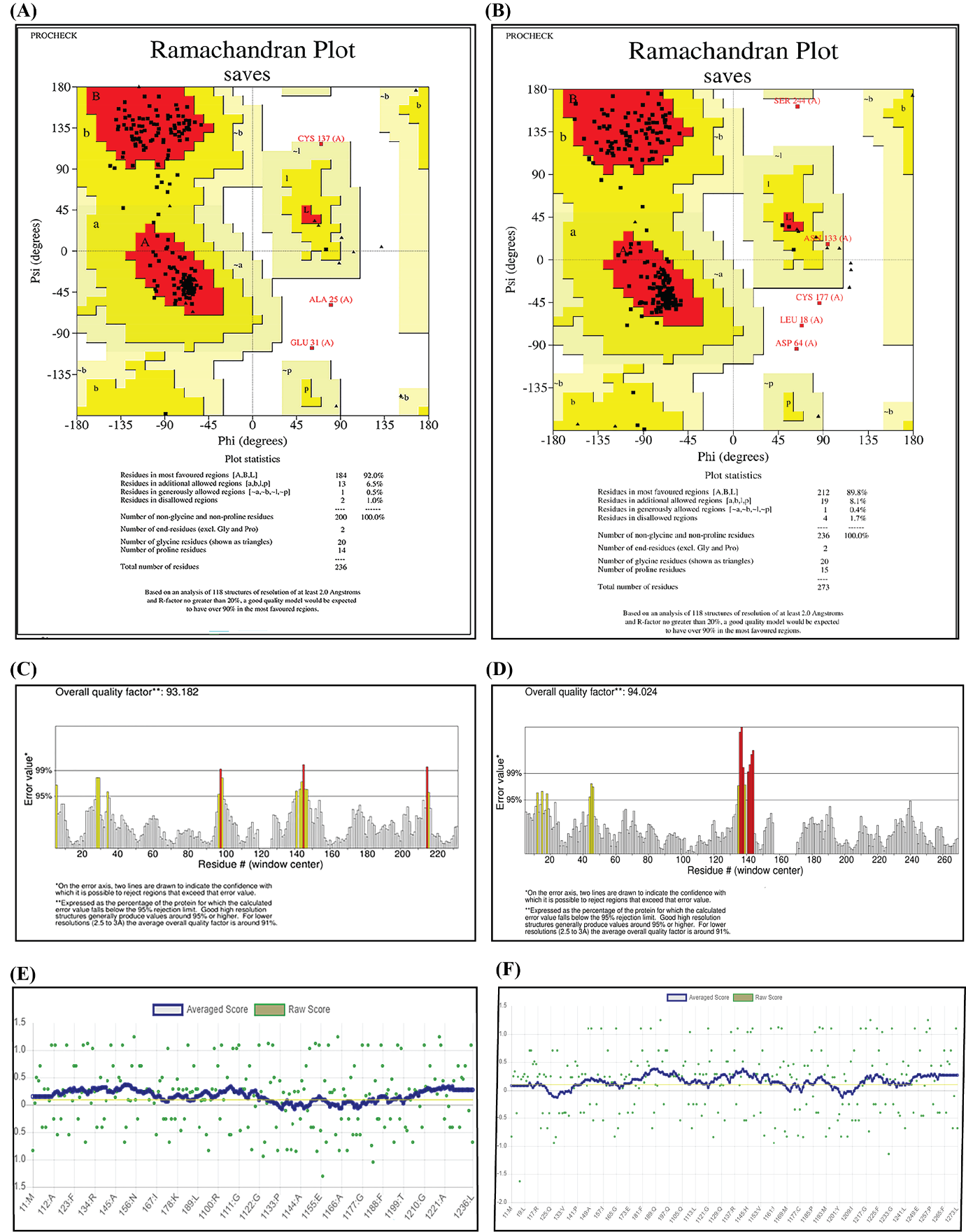


**Figure S4.**  Ramachandran plot analysis of the SeCobB_S_ (A) and SeCobB_L_ (B) modelled structure after validation. Quality factor analysis of SeCobB_S_ (C) and SeCobB_L_ (D) modelled structure by ERRAT server. Compatibility of the predicted modelled structure of SeCobB_S_ (E) and SeCobB_L_ (F) with its amino acid sequence using VERIFY3D server.

**
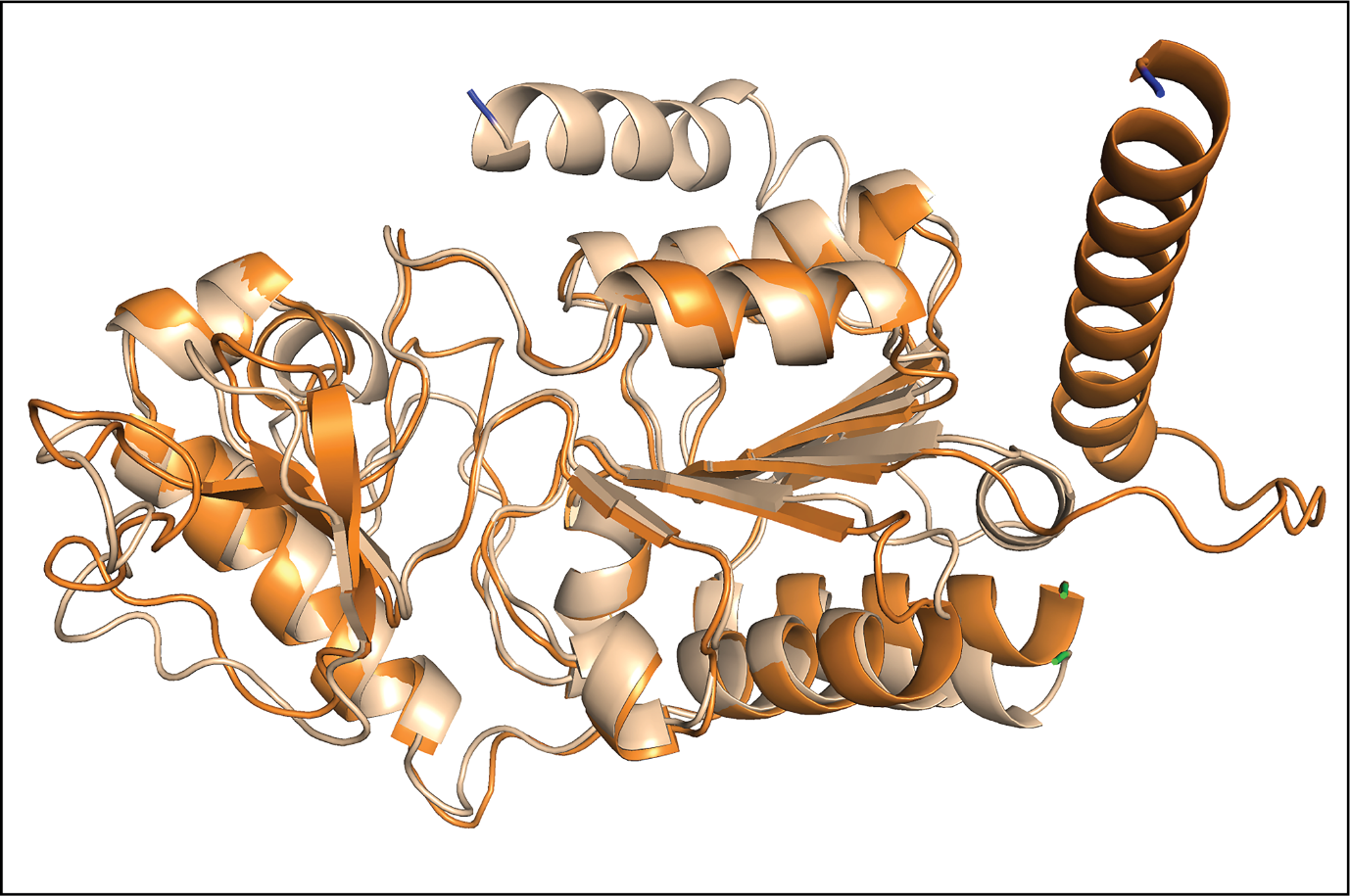
**

**Figure S5:** Cartoon representation of the superimposed modeled (I-TASSER server) longer isoform (wheat in color) with Alphafold modeled structure (Orange in color) of NAD^+^-dependent protein deacylase of *Salmonella* enteritidis PT4 (strain P125109). The N-terminal and C-terminal ends are shown in blue and cyan color respectively. The RMSD between the two structures was found to be 1.549 Å.


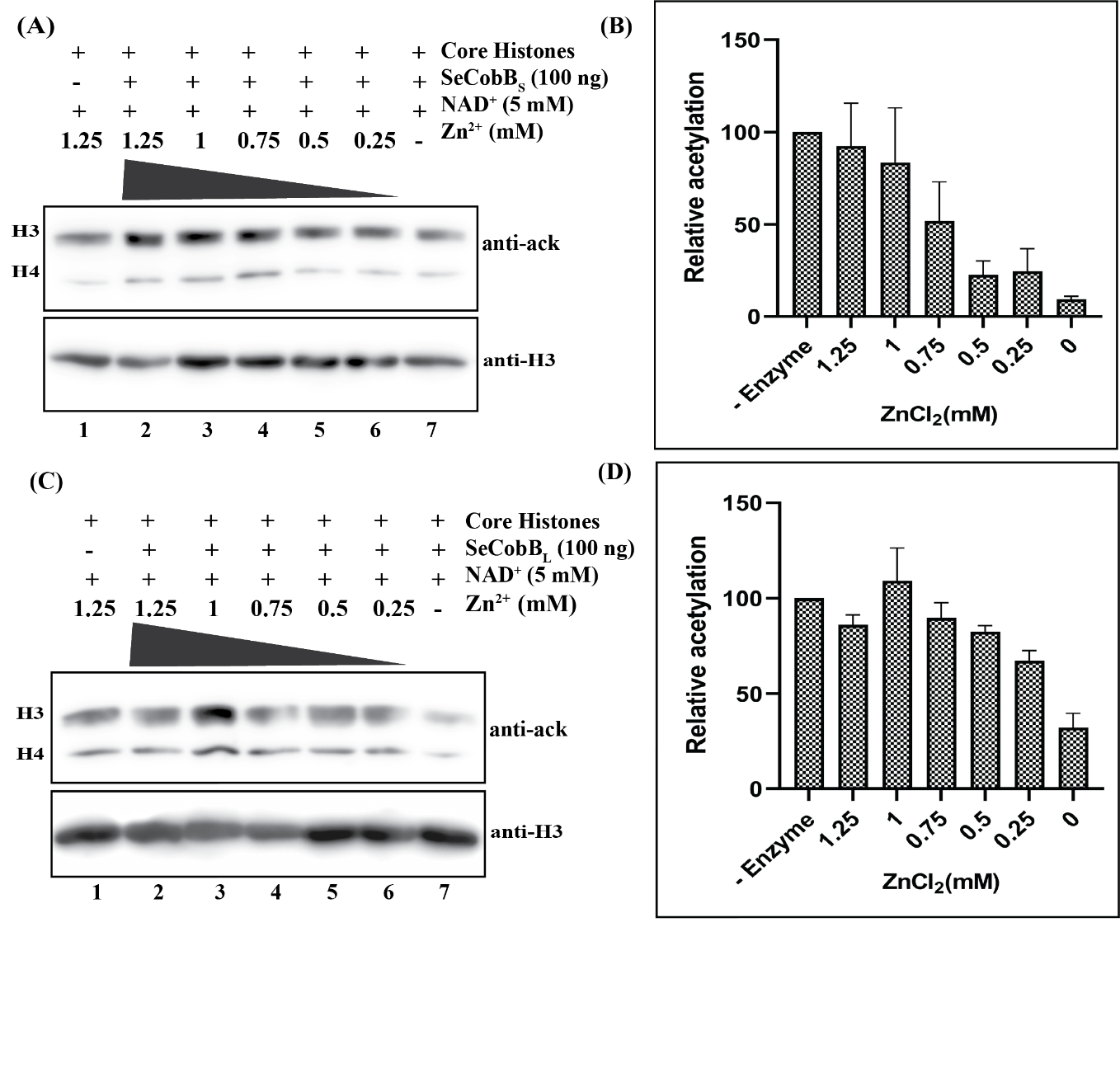


**Figure S6:**  The relative intensity graph of SeCobB_S_ (B) and SeCobB_L_ (D) corresponding to their respective inhibition in deacetylase activity in the presence of ZnCl_2,_ respectively_._ (A & B) Figure A and B is figure 3A and 3B in the main text. The % relative intensity represents level of inhibition in the enzyme activity as compared to without ZnCl_2_ lane.


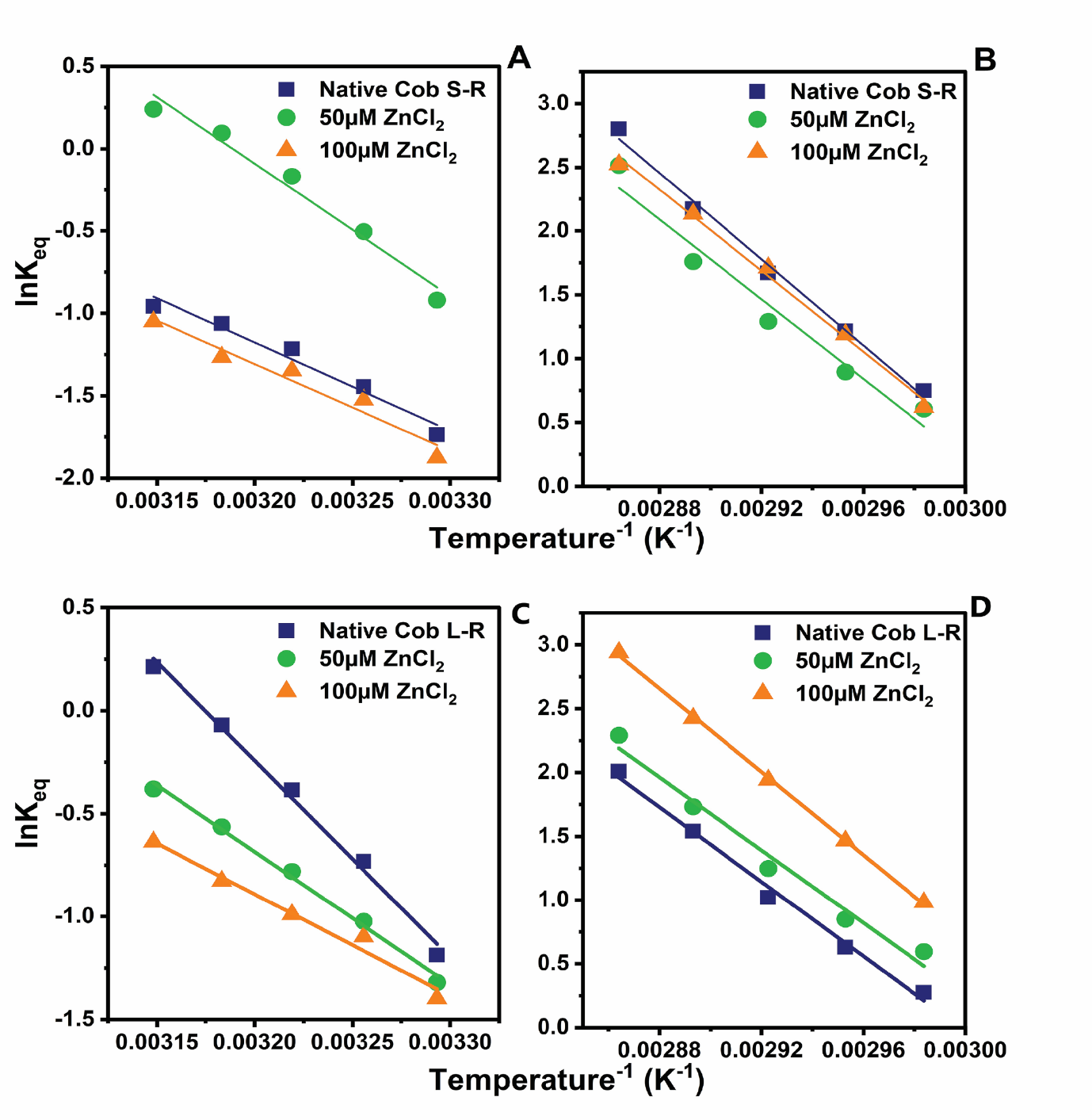


**Figure S7: Van't Hoff plots for the data shown in Figure 6.** The values of van't Hoff enthalpy (ΔH_VH_) are estimated with the aid of equation as described previously i.e. ${lnK}_{eq (folded\to unfolded)}=\frac{{-\Delta H}_{vH}}{RT}+ \frac{\Delta S}{R}$ [[2](#_ENREF_3)] in the presence and absence of variable concentration of zinc ions**.**

**References**

1. Robert, X. and P. Gouet, *Deciphering key features in protein structures with the new ENDscript server.* Nucleic Acids Res, 2014. **42**(Web Server issue): p. W320-4.

2. Nandi, S.K., et al., *A S52P mutation in the ‘α-crystallin domain’ of Mycobacterium leprae HSP18 reduces its oligomeric size and chaperone function.* The FEBS Journal, 2013. **280**(23): p. 5994-6009.
